# Innovative Therapeutic and Delivery Approaches Using Nanotechnology to Correct Splicing Defects Underlying Disease

**DOI:** 10.3389/fgene.2020.00731

**Published:** 2020-07-14

**Authors:** Marc Suñé-Pou, María J. Limeres, Cristina Moreno-Castro, Cristina Hernández-Munain, Josep M. Suñé-Negre, María L. Cuestas, Carlos Suñé

**Affiliations:** ^1^Drug Development Service (SDM), Faculty of Pharmacy, University of Barcelona, Barcelona, Spain; ^2^Institute of Research in Microbiology and Medical Parasitology (IMPaM), Faculty of Medicine, University of Buenos Aires-CONICET, Buenos Aires, Argentina; ^3^Department of Molecular Biology, Institute of Parasitology and Biomedicine “López-Neyra” (IPBLN-CSIC), Granada, Spain; ^4^Department of Cell Biology and Immunology, Institute of Parasitology and Biomedicine “López-Neyra” (IPBLN-CSIC), Granada, Spain

**Keywords:** splicing, RNA, gene therapy and therapeutic delivery, siRNAs, ASOs, SMaRT, gene editing, nanoparticle

## Abstract

Alternative splicing of pre-mRNA contributes strongly to the diversity of cell- and tissue-specific protein expression patterns. Global transcriptome analyses have suggested that >90% of human multiexon genes are alternatively spliced. Alterations in the splicing process cause missplicing events that lead to genetic diseases and pathologies, including various neurological disorders, cancers, and muscular dystrophies. In recent decades, research has helped to elucidate the mechanisms regulating alternative splicing and, in some cases, to reveal how dysregulation of these mechanisms leads to disease. The resulting knowledge has enabled the design of novel therapeutic strategies for correction of splicing-derived pathologies. In this review, we focus primarily on therapeutic approaches targeting splicing, and we highlight nanotechnology-based gene delivery applications that address the challenges and barriers facing nucleic acid-based therapeutics.

## Introduction

The genome is the complete set of DNA that contains all the information necessary for the development and survival of an organism. In humans, the genome is contained in 23 chromosome pairs, comprising approximately 21,000 protein-coding genes and slightly over 3 billion DNA base pairs in total. Although the human genome was sequenced approximately fifteen years ago, there is still disagreement regarding the number of genes due to inconsistencies in the available databases (Willyard, 2018). Regardless of the final count, the numbers of protein-coding genes are similar between humans and worms (∼21,000 and ∼19,000, respectively), and the number found in flies is not drastically lower (∼14,000); however, humans are more complex than these other organisms. The explanation for this apparent paradox may be related to the large predicted number of proteins encoded by the human genome (possibly more than 100,000) as a result of regulatory processes such as alternative transcription, splicing, 3′-end formation, translation and posttranslational modifications. To date, there is no solid evidence (based on, for example, mass spectrometry) to support the existence of this level of complexity in the human proteome. Thus, ambitious projects are being designed to identify and characterize protein variant isoforms for each protein-coding gene (Baker et al., 2017).

Alternative splicing (AS), the phenomenon by which a single precursor (pre-) messenger RNA (mRNA) can generate alternative mRNAs to yield proteins with related or different functions, expands the protein information encoded by the genome. Global transcriptome analyses have estimated that 95–100% of multiexon genes undergo AS (Pan et al., 2008; Wang et al., 2008). The best-known example of the considerable transcriptome diversity resulting from this process is the *Drosophila* Down syndrome cell adhesion molecule (*Dscam*) gene, which may give rise to 38,016 cell-surface proteins through AS (Wojtowicz et al., 2007). The functional genome-wide consequences of AS are exemplified by the finding that distinct alternative isoforms encoded by a single gene exhibit distinct protein interaction profiles (Yang et al., 2016). Each of these protein isoforms can be further processed through posttranslational modifications to yield many more distinct proteoforms (Smith et al., 2013) harboring new functions. Recent technological and bioinformatics advances will help to unambiguously decipher the specific sequence and amount of each RNA molecule synthesized by a given cell (Hardwick et al., 2019). In addition, AS approaches specifically designed for single-cell RNA sequencing (scRNAseq) data are emerging, and these approaches may greatly improve our understanding of isoform usage at the single-cell level (Chen et al., 2019).

Alternative splicing in eukaryotes has considerable impacts on a variety of biological pathways; therefore, it is not surprising that AS is a highly orchestrated process involving multiple protein–protein and protein–RNA interactions. The spliceosome, the multiprotein complex that performs the splicing reaction, is composed of a core of five uridine-rich small nuclear RNAs (snRNAs; termed U1, U2, U4, U5, and U6) and 200 other proteins. The spliceosome assembles on pre-mRNA to remove noncoding introns through two sequential transesterification reactions: the branching and exon ligation steps. Detailing all the steps of the AS process is beyond the scope of this review, and comprehensive reviews on the molecular choreography of pre-mRNA splicing have been published elsewhere (Wahl et al., 2009; Matera and Wang, 2014; Wan et al., 2019). In this review, we briefly summarize some aspects of splicing regulation before turning toward advances in therapies and nanodelivery systems targeting splicing for the treatment of human disease.

**FIGURE 1:**
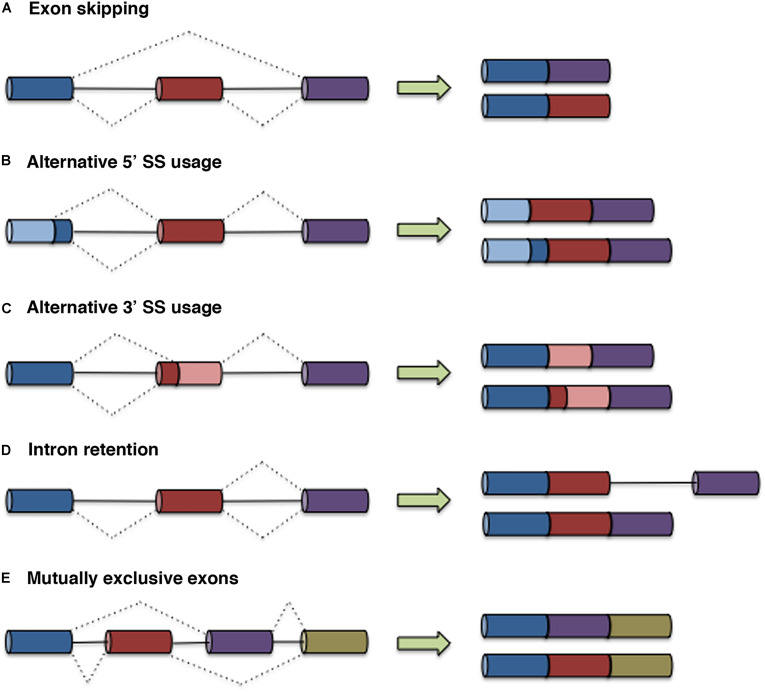
Types of AS. In the graphs, exons are represented by boxes, and introns are represented by lines. Dashed lines indicate AS events. The five main types of AS are illustrated: exon skipping **(A)**, alternative 5′ SS usage **(B)**, alternative 3′ SS usage **(C)**, intron retention **(D)**, and mutually exclusive exons **(E)**.

The major forms of AS include exon skipping, alternative 3′ and 5′ splice site (SS) usage, intron retention, and mutual exon exclusion (Figure 1). Other events that generate different transcript isoforms include alternation of initial exons due to alternative promoter usage and alternation of terminal exons due to alternative polyadenylation. Recognition of exon/intron boundaries for correct intron removal by the splicing machinery requires the presence of several sequence elements on pre-mRNA, including the 5′ and 3′ SSs, the branch point sequence (BPS), and the polypyrimidine (Py) tract. In addition to these core SS motifs, other *cis*-regulatory elements that recruit specific RNA-binding proteins that either activate or repress the use of adjacent SSs contribute to the fine-tuning and specificity of this pre-mRNA processing event. These sites, known as exonic splicing enhancers (ESEs) or silencers (ESSs) and intronic splicing enhancers (ISEs) or silencers (ISSs), recruit specific *trans*-acting proteins such as heterogeneous nuclear ribonucleoproteins (hnRNPs) and serine/arginine (SR) proteins (Wu and Maniatis, 1993). AS can also be regulated at the levels of transcription and chromatin structure, adding complexity to the molecular mechanisms that govern splicing control. Two models have been proposed to explain the link between transcription and splicing. The first model, known as the recruitment model, involves the recruitment of splicing factors to pre-mRNA through RNA polymerase II (RNA Pol II) (McCracken et al., 1997). In the second model, the kinetic model, the transcript elongation rate influences AS (de la Mata et al., 2003; Montes et al., 2012; Dujardin et al., 2014; Fong et al., 2014). Chromatin structure, DNA methylation, histone marks, and nucleosome positioning also impact AS by affecting transcription and/or cotranscriptional splicing. An excellent review covering the different levels at which AS is regulated has been published previously (Naftelberg et al., 2015).

## AS and Disease

**FIGURE 2:**
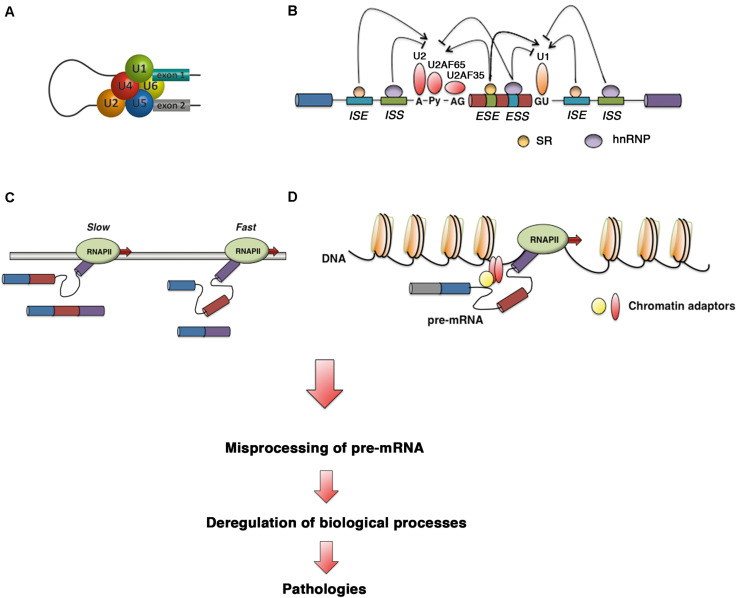
Defective control in pre-mRNA splicing leads to human diseases. Diagram showing how defects in **(A)** core spliceosome components, **(B)** *trans*-acting splicing regulatory factors and *cis*-regulatory signals, **(C)** the transcription rate, and **(D)** chromatin structure or marks can disrupt splicing regulation to cause human disease via misprocessing of pre-mRNA.

Defects in core spliceosome components, *trans*-acting splicing regulatory factors, *cis*-regulatory signals, and the transcription rate or changes in chromatin structure or marks can cause multiple pathologies as a result of misprocessing of pre-mRNA, highlighting the importance of RNA processing (Figure 2). To date, 23,868 mutations responsible for human inherited disease that have been reported in the Human Gene Mutation Database (HGMD, accessed in February 2020) have consequences for mRNA splicing (accounting for 8.7% of heritable disease-causing mutations) (Stenson et al., 2012). This number is likely an underestimate, since most of the reported mutations have been identified by genomic DNA sequencing without consideration that missense, nonsense and synonymous changes can affect splicing, as reported previously for coagulation factor IX exon 5 (Tajnik et al., 2016). It has been estimated that up to 50% of mutations that lead to heritable disease occur as a result of errors in the RNA splicing process or its regulation (Lopez-Bigas et al., 2005; Taylor and Lee, 2019). While we wait for functional testing of predicted mutations, the development of new and effective predictive algorithms for splicing effect analysis is critical (Anna and Monika, 2018).

Recent advances in the development of novel technologies and tools, such as next-generation sequencing and clustered regularly interspaced short palindromic repeats (CRISPR)/CRISPR-associated protein-9 nuclease (Cas9) genome editing technology, have greatly expanded the available information regarding RNA missplicing events and their association with diseases. These technologies have demonstrated the presence of many naturally occurring genetic variants that affect AS and lead to phenotypic variability and disease susceptibility among humans (Park et al., 2018). For example, 371 splicing quantitative trait loci (sQTLs), including sQTLs in known type 2 diabetes-associated genes or in genes associated with beta cell function and glucose metabolism in human pancreatic islets, have been identified; these sQTLs may aid in elucidation of individual susceptibility to type 2 diabetes (Fadista et al., 2014). Recently, Takata et al., analyzed RNA-seq data on human brain tissues from more than 200 individuals in combination with genotype data and identified approximately 1,500 sQTLs throughout the genome. Interestingly, these researchers observed significant enrichment of epigenetic mark variants that may influence transcriptional activation and AS. In a comparative analysis of genome-wide association study (GWAS) data, many of the observed variants were found to be associated with various human diseases, particularly schizophrenia (Takata et al., 2017).

Mutations that occur in genes encoding fundamental components of the splicing machinery have been described in many splicing-related diseases. However, the frequency of these mutations is low, probably because their effects are incompatible with life. Disease-associated mutations that occur within introns lead to intron retention or to exon skipping upstream or downstream of the mutated SSs without affecting the coding sequence. In contrast, exonic mutations may or may not affect the coding sequence depending on the type of mutation (silent *versus* missense or nonsense) and can also alter the splicing pattern. Thus, mutations in introns or exons may disrupt RNA secondary structure or disrupt or create *de novo* cryptic SSs or *de novo* splicing silencers and enhancers, leading to dysregulation of AS. Mutations or quantitative changes in proteins with regulatory functions during the splicing process can also lead to aberrant splicing, affecting many RNA transcripts at the same time (Havens et al., 2013).

With regard to cancer, genomic studies have identified frequent and recurrent mutations in genes that code for pre-mRNA splicing factors in both hematological malignancies (e.g., myelodysplastic syndrome [MDS], acute myeloid leukemia, and chronic lymphocytic leukemia) (Yoshida et al., 2011) and solid malignancies (e.g., breast cancer, lung cancer, pancreatic cancer and uveal melanoma) (Imielinski et al., 2012; Harbour et al., 2013; Bailey et al., 2016; Nik-Zainal et al., 2016). These findings suggest a potential relationship between certain spliceosome gene mutations and carcinogenesis. For MDS, *SF3B1*, *SRSF2*, *U2AF1*, and *ZRSR2* are the four most commonly mutated splicing factor genes, although mutations in other splicing factor genes have also been observed (Taylor and Lee, 2019). Although the underlying mechanisms and contributions of splicing factors in cancer pathogenesis have not been elucidated, and although more work is needed to understand the splicing alterations observed in cancer cells, these data identify novel opportunities for development of splicing-based cancer therapies.

Recent advances in the treatment of some diseases have led to improvements in patient prognosis and life expectancy. For example, spinal muscular atrophy (SMA) type 1, which is considered to be most serious at an early age, can currently be treated with Zolgensma^®^. Zolgensma^®^ is a new gene therapy-based drug approved by the United States Food and Drug Administration (FDA) that improves the quality of life and life expectancy of infants with SMA type 1. Although this treatment can cure this deadly inherited disease, the Swiss multinational corporation Novartis AG has established a sale price of 2.1 million dollars for a single intravenous administration. This drug is by far the most expensive pharmacological treatment in existence today.

Identification of splicing mutations has significantly advanced our understanding of how splicing dysregulation contributes to disease pathogenesis and of how splicing, a key pre-mRNA processing event, can be targeted for therapeutic applications. In Supplementary Table 1, we provide a list of the most frequent splicing-related human diseases that could be targeted for gene therapy. To learn more, readers are directed to several comprehensive reviews covering human diseases caused by RNA missplicing that have been published elsewhere (Cieply and Carstens, 2015; Daguenet et al., 2015; Chabot and Shkreta, 2016; Scotti and Swanson, 2016).

## Therapeutic Approaches

Designing effective therapeutic strategies to overcome the consequences of aberrant splicing events on disease states remains a major challenge. Gene therapy has emerged as a promising pharmacotherapeutic option for patients with diseases of genetic origin. Hence, targeting of aberrant RNA splicing is a logical approach for directly correcting disease-associated splicing alterations without affecting the genome. Other approaches, such as targeting splicing reactions to disrupt the expression of disease-related proteins or targeting exon junctions mutated mRNA to disrupt protein coding, can be used to reframe and rescue protein expression (Havens et al., 2013).

**FIGURE 3:**
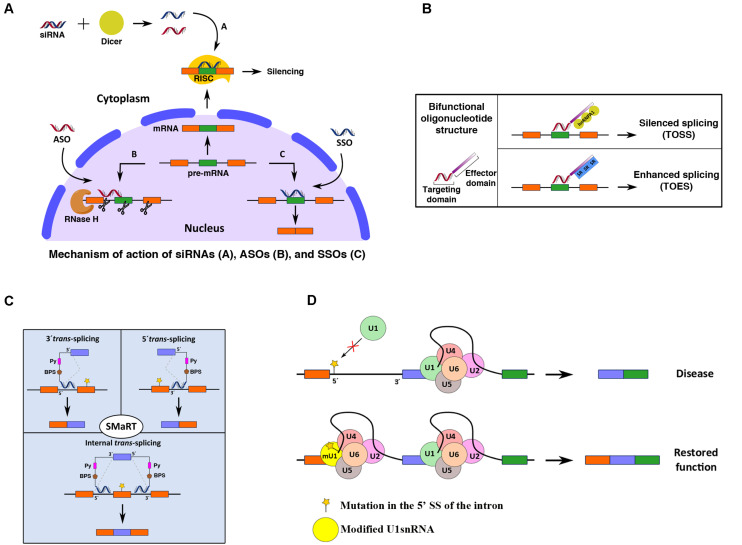
Schematic representation of different splicing-targeting strategies for gene modification. **(A)** siRNA-, ASO-, and SSO-based strategies (a, b, and c in the figure, respectively) for pre-mRNA editing. **(B)** Bifunctional oligonucleotides contain one small sequence complementary to the pre-mRNA (targeting domain) and another region (effector domain) that recruits specific regulatory factors to modulate the splicing outcome (TOSS and TOES). **(C)** Three types of SMaRT approaches are depicted: 5′ *trans*-splicing, 3′ *trans*-splicing, and IER, which target the 5′ end, 3′ end, or internal portion of a mutated target pre-mRNA, respectively. See the text for further details on these RNA splicing-editing mechanisms. **(D)** Schematic representation of the general strategy for correction of splicing defects using modified snRNAs. The cartoon shows the restoration of a mutated 5′ SS of an intron using a modified U1 snRNA.

Several strategies have been designed to manipulate the splicing process, including spliceosome-mediated RNA *trans*-splicing (SMaRT) and the use of antisense oligonucleotides (ASOs), bifunctional oligonucleotides, small-molecule compounds, and modified snRNAs (Figure 3). All of these approaches have been used to correct the effects of RNA misprocessing. In recent years, genome editing techniques involving zinc finger (ZF) proteins (ZFPs), transcription activator-like nucleases (TALENs) and CRISPR/Cas9 systems have become new treatment avenues for correction of splicing defects (Hsu et al., 2014; Fernandez et al., 2017; Knott and Doudna, 2018).

### RNA Editing Approaches

#### Antisense Oligonucleotides (ASOs)

Antisense oligonucleotides strategies use short synthetic single-stranded DNA molecules that are complementary to a specific pre-mRNA sequence to alter the splicing process. ASO binding to a target pre-mRNA in the nucleus sterically blocks the recruitment of *trans*-acting proteins to the pre-mRNA sequence at the target site (Figure 3A).

ASOs can be designed to target (i) the SS, thereby redirecting splicing to an adjacent site; (ii) auxiliary sequences (enhancers or silencer elements) within the immature transcript, thereby modifying the outcome of the splicing reaction (by blocking or promoting splicing); and (iii) RNA bases, thereby stabilizing/destabilizing regulatory structures and modifying the splicing outcome (Havens et al., 2013; Sune-Pou et al., 2017).

Splicing-related ASOs that act according to the first two mechanisms mentioned above by promoting or redirecting splicing are also called splice-switching oligonucleotides (SSOs). These short, 15- to 30-nucleotide-long sequences sterically block important motifs in pre-mRNA (i.e., SSs and/or regulatory sequences) to prevent RNA–RNA base-pairing or protein–RNA binding interactions between spliceosome components and pre-mRNA without promoting degradation of the RNA transcript while altering splicing outcomes (Havens and Hastings, 2016). The nucleotides of an SSO are chemically modified (e.g., into morpholino antisense oligomers) such that the RNA-cleaving enzyme RNase H is not recruited to degrade the pre-mRNA–SSO complex (Summerton, 1999). This property differs from the RNase H activity exerted by conventional ASOs, which inhibits gene expression by degrading the target pre-mRNA. Chemical modifications of ASOs are also crucial because they stabilize the ASOs *in vivo* and improve their cellular uptake, release and binding affinity for their targeted RNA sequences; unmodified oligonucleotides are highly susceptible to degradation by circulating nucleases and are excreted by the kidneys. Examples of these chemical modifications include changes to the phosphate backbones and/or sugar components of the oligonucleotides, such as the use of a phosphorothioate backbone (first-generation ASOs) (Eckstein, 2014), the use of locked nucleic acid chemistry for bridging of the sugar furanose ring (Campbell and Wengel, 2011), alterations at 2′ positions of the ribose sugar ring (2′-O-methylation [2′-OMe] and 2′-O-methoxyethylation [2′-MOE]) (second-generation ASOs) (Geary et al., 2001), and the addition of phosphorodiamidate morpholinos (third-generation ASOs) (Summerton, 1999).

The clinical application of this technology has resulted in the commercialization of Vitravene^TM^ (fomivirsen), which, in 1998, became the first ASO approved by the FDA for the treatment of AIDS-related cytomegalovirus retinitis; Macugen^TM^ (pegaptanib), approved by the FDA in 2004 for the treatment of neovascular age-related macular degeneration; and Kynamro^TM^ (mipomersen), approved by the FDA in 2013 for the treatment of homozygous familial hypercholesterolemia. These products have been withdrawn from the market for commercial reasons owing to an overall small patient population and competing alternative drugs, such as statins in the case of familial hypercholesterolemia (Sharma and Watts, 2015). The first published report on the use of ASOs as a splicing-targeting therapeutic tool was published by Dominski and Kole in 1993. These authors restored correct splicing in thalassemic pre-mRNA by using a 2′-OMe ASO (Dominski and Kole, 1993). Since then, many ASO strategies have been designed to modify splicing for the treatment of several diseases, and some of them are currently in clinical trials. Only two ASOs are already FDA approved (Supplementary Table 2). Exondys 51^TM^ (eteplirsen) was the first drug in its class to be approved by the FDA (in September 2016) under the Accelerated Approval Program to treat patients with Duchenne muscular dystrophy (DMD). Exondys 51^TM^ belongs to the third generation of phosphorodiamidate morpholino ASOs and is specifically indicated for patients who have a confirmed mutation of the dystrophin gene amenable to exon 51 skipping. This mutation affects approximately 13% of the population with DMD. Recently, Vyondys 53^TM^ (golodirsen), a DMD drug that is highly similar to eteplirsen except that involves exon 53 skipping rather than exon 51 skipping, was denied by the FDA in August 2019 because of the risk of infections related to intravenous infusion ports and renal toxicity seen in preclinical studies. A similar decision was reached in January 2016 for the promising drug Kyndrisa^TM^ (drisapersen). This drug was intended for the treatment of patients with DMD amenable to exon 51 skipping but failed to demonstrate substantial effectiveness. The pharmaceutical company BioMarin invested over $66 million in the development of drisapersen; as a consequence of the denial, BioMarin also discontinued the development of three follow-on products of drisapersen, BMN 044, BMN 045, and BMN 053. These products were in mid-stage trials for specific forms of the muscle-wasting disease.

In December 2016, the FDA approved Spinraza^TM^ (nusinersen), the first drug approved for the treatment of SMA the first drug approved for the treatment of SMA (Lorson et al., 1999; Monani et al., 1999). Spinraza^TM^ is a 2′-OMe phosphorothioate ASO that targets an intron 7 internal SS within *SMN2* pre-mRNA, inducing exon 7 inclusion and producing a functional SMN protein. Notably, because ASOs generally do not cross the blood–brain barrier, repeated intrathecal nusinersen delivery is required. This requirement is highly disadvantageous and makes administration challenging, especially for infants (Verma, 2018).

Investigational ASOs for Huntington’s disease (HD), amyotrophic lateral sclerosis (ALS) and transthyretin (TTR) amyloidosis, such as RG6042, tofersen and inotersen, respectively, are currently in phase III clinical trials. RG6042 (previously known as IONIS-HTTRx) reduces the concentration of mutant huntingtin (HTT) levels in the cerebrospinal fluid of patients with HD without causing serious adverse events (Tabrizi et al., 2019). Tofersen (previously known as BIIB067) targets superoxide dismutase (SOD1) in ALS patients, reducing SOD1 concentrations in spinal fluid to preserve motor neurons and slow the progression of the disease. Inotersen is a 2′-MOE-modified ASO that reduces the production of TTR and improves disease course and quality of life in early hereditary TTR amyloidosis polyneuropathy (ATTR) (Mathew and Wang, 2019).

The development of the ASO milasen is an example of how cutting-edge medicine can be used with great speed for patient-customized treatment of a rare and fatal neurodegenerative disease (ceroid lipofuscinosis 7, CLN7, a form of Batten’s disease). Researchers at Boston Children’s Hospital identified a novel mutation in a 6-year-old girl, designed and produced an ASO, and obtained FDA approval for its clinical deployment in less than one year (Kim et al., 2019).

ASOs are also applicable to cancer treatment. For instance, Dewaele et al., used ASO-mediated exon skipping to decrease the expression of *MDM4*, a splice isoform produced in cancer cells (Dewaele et al., 2016). Similarly, Hong et al. preclinically and clinically evaluated a chemically modified ASO termed AZD9150 that targets the *STAT3* gene, a transcriptional activator and oncogenic mediator of the JAK-STAT signaling pathway (Hong et al., 2015). Ross et al. described the use of an ethyl-containing ASO (AZD4785) to downregulate *KRAS* mRNA, which is mutated in approximately 20% of human cancers, and demonstrated its efficacy in preclinical *KRAS* mutant lung cancer models (Ross et al., 2017).

Antisense oligonucleotides have been validated as therapeutic agents; however, because of the high cost associated with these products, improvements must be made to prevent health insurance companies from denying patients access. For instance, Exondys 51^TM^ is priced at $300,000 per patient per year even though the efficacy of this drug is controversial.

#### Bifunctional Oligonucleotides

Bifunctional oligonucleotides are ectopic modulators of AS used to control the patterns of splicing of specific genes. In brief, these oligonucleotides contain two parts: (i) an antisense portion targeting a specific sequence and (ii) a nonhybridizing tail or effector domain that recruits acting factors (targeted oligonucleotide enhancer of splicing [TOES] or targeted oligonucleotide silencer of splicing [TOSS]) (Brosseau et al., 2014) (Figure 3B). Oligonucleotides containing binding site sequences for the splicing repressor hnRNP A1/A2 have been used to reprogram AS via TOSS. The oligonucleotides were positioned upstream of a 5′ SS to interfere with U1 snRNP binding and repress SS use (Villemaire et al., 2003). Dikson et al. used a TOSS with hnRNPA1 tails to block the inclusion of exon 8 in *SMN2*, thereby favoring exon 7 inclusion and restoring the functionality of the protein (Dickson et al., 2008). Similarly, bifunctional TOES, whose tail of enhancer sequences recruits activating proteins such as positively acting SR proteins, has been used to increase the splicing of refractory exon 7 in *SMN2* in fibroblasts derived from patients with SMA (Skordis et al., 2003; Owen et al., 2011). This approach is mechanistically different than ASO approaches, although both can be used for stimulating the inclusion of exon 7 in SMA. Whereas ASOs such as Spinraza^TM^ block the binding of splicing factors to intron 7, causing exon 7 inclusion, bifunctional TOSS/TOES is intended to direct exon 7 inclusion and thus restore protein expression.

#### Small Interfering RNAs (siRNAs)

In 1998, another related technology emerged with the discovery of the siRNA pathway, which can be used to silence the expression of genes (Fire et al., 1998). The discovery of RNA interference (RNAi), in which double-stranded RNA (dsRNA) is hybridized with a specific mRNA sequence to induce its silencing or degradation, was a major scientific breakthrough for which Craig Mello and Andrew Fire were recognized with the Nobel Prize in Physiology and Medicine in 2006. This discovery revolutionized the way scientists study gene function and offered an innovative strategy for the treatment of diseases, particularly those of genetic origin, as demonstrated for the first time by Elbashir et al. (2001). Through this strategy, administration of synthetic 21- to 25-nucleotide duplexes with overhanging 3′ ends (siRNAs) can be used to suppress the expression of endogenous and heterologous genes (Figure 3A). Although many classes of small RNAs have emerged, three main categories are widely recognized: siRNAs; microRNAs, or miRNAs; and piwi-interacting RNAs, or piRNAs. These RNA types differ in structure, biological roles, associated effector proteins and origins (Dana et al., 2017). Physiologically, in cells, siRNAs help to maintain genomic integrity by preventing the action of foreign nucleic acids, including those of viruses, transposons and retrotransposons and transgenes, while miRNAs act as posttranscriptional endogenous gene regulators (Meister and Tuschl, 2004). piRNAs have been implicated in the silencing of retrotransposons at both the posttranscriptional and epigenetic levels as well as other genetic elements in germlines, particularly those activated during spermatogenesis (Siomi et al., 2011). Upon delivery into cells, siRNAs are bound by a multiprotein component complex, known as the RNA-induced silencing complex (RISC), in the cytoplasm; the two strands are then separated, and the strand with the RISC hybridizes with the target mRNA. After that step, Argonaute-2 (Ago2), a catalytic component of the RISC, drives mRNA cleavage (Dana et al., 2017). Since siRNA-mediated targeting of aberrant splicing isoforms is widely used as an RNAi technology, only siRNA approaches will be discussed in detail in this section (Figure 3A).

These siRNA approaches can be used to target aberrant splicing isoforms for therapeutic applications (Sune-Pou et al., 2017). Indeed, siRNAs targeting exonic/intronic sequences close to alternative exon or exonic/intronic junction sequences can induce degradation of alternatively spliced and aberrant mRNAs without affecting the expression of normal mRNAs. Such targeting approaches have been used for diseases such as Ullrich congenital muscular dystrophy (UCMD) (Bolduc et al., 2014), growth hormone deficiency (GHD) type II diseases (Ryther et al., 2004) and several cancers. In the context of cancer, it has been observed that the occurrence of specific splice variants is increased during tumorigenesis and that the splicing regulatory machinery is abnormal in many malignant cells (Hayes et al., 2006). Bolduc et al. designed different siRNAs targeting the most frequent mutation that causes exon 16 skipping in the *COL6A3* gene and tested the siRNAs *in vitro* in UCMD-derived dermal fibroblasts. These siRNAs resulted in specific knockdown of the mutant allele and increased the abundance and quality of collagen VI matrix production (Bolduc et al., 2014). Similarly, Ryther et al. used siRNAs to specifically degrade exon 3-skipped transcripts in a GHD type II disease (Ryther et al., 2004). Finally, Hayes et al. have shown that siRNA-mediated downregulation of SR protein kinase 1 (SRPK1), which is significantly upregulated in tumors of the pancreas, breast and colon, decreases cell proliferation and increases apoptosis. Moreover, the sensitivity of tumor cells to chemotherapeutic agents such as gemcitabine and cisplatin increases upon treatment with this siRNA (Hayes et al., 2006).

In October 2016, the development of revusiran, an siRNA designed for the treatment of ATTR amyloidosis, was suspended after a randomized double-blind placebo-controlled phase III trial of its efficacy and safety, ENDEAVOUR, demonstrated that the siRNA caused greater mortality than a placebo (18 of 206 enrolled patients). However, in August 2018, the FDA approved Onpattro^TM^ (patisiran), the first siRNA-based drug for the treatment of hereditary TTR-mediated amyloidosis (hATTR) (Supplementary Table 2). Hence, siRNA-based technology has shown promising therapeutic results and the possibility of translation into clinical use.

#### Small-Molecule Compounds

Small-molecule compounds can also be used to modulate RNA expression. Some molecules are capable of binding specific three-dimensional RNA structures, thereby preventing their translation or function. Furthermore, these compounds can also modify splicing factor activity (by affecting posttranslational modifications of splicing factors) or directly alter splicing events. Compared with oligonucleotide-based therapeutics, these compounds are easier to deliver to target sites and normally have lower toxicity profiles. However, small-molecule compounds frequently act through unknown mechanisms, resulting in a lack of information, and have less target specificity than other therapeutic formulations, thus potentially exhibiting more nonspecific and off-target effects. While oligonucleotides (ASOs, TOSS, TOES and siRNAs) can very specifically and efficiently modulate particular RNA targets upon complementary recognition of the RNA sequences, small molecules can recognize specific three-dimensional structures and overcome known oligonucleotide drawbacks, e.g., poor pharmacological properties.

Some small molecules have already been approved for use in clinical practice for applications other than splicing defect correction. For example, digoxin and other prescribed cardiotonic steroids, routinely used in the treatment of heart failure, have been described as modulators of AS (Stoilov et al., 2008). Pentamidine and Hoechst 33258 also modulate AS in myotonic dystrophy (DM) by disrupting MBNL1 binding to CUG *in vitro* and *in vivo* (Warf et al., 2009; Parkesh et al., 2012). The histone deacetylase sodium butyrate, which is known to upregulate the expression of splicing factors, has been demonstrated to increase *CFTR* transcript levels, leading to activation of CFTR channels and restoration of their function in CFTR-derived epithelial cells (Nissim-Rafinia and Kerem, 2006). It has also been reported that the plant cytokinin kinetin improves *IKBKAP* mRNA splicing in patients with familial dysautonomia (FD) (Axelrod et al., 2011).

Some small-molecule splicing modulators have been evaluated in clinical trials for the treatment of solid tumors and leukemia. For instance, E7107 (pladienolide B) is a splicing modulator whose preferential cytotoxicity is positively influenced by some antiapoptotic *BCL2* family genes, such as *BCL2A1*, *BCL2L2* and *MCL1*. Furthermore, Aird et al. have reported that combinations of E7107 and *BCLxL* (*BCL2L1*-encoded) inhibitors enhance cytotoxicity in cancer cells (Aird et al., 2019). Notably, E7107 was the first compound of a new class of anticancer agents targeting the spliceosome. Specifically, E7107 interacts with subunit 1 of SF3b to block the normal splicing of oncogenes. Unfortunately, the development of E7107 was suspended after phase I clinical trials due to an unacceptable profile of adverse events. Herboxidienes, spliceostatin A, meayamycin B and sudemycin D6/K have also been shown to exhibit antitumoral activity *in vivo* by targeting the SF3b subunit of the spliceosome (Lin, 2017). Recently, Carabet et al. reported VPC-80051 as the first small-molecule inhibitor of hnRNPA1, which plays an important role in cancer by controlling the transcriptional levels of the oncoprotein c-Myc (Carabet et al., 2019). Thus, small molecules that selectively inhibit hnRNP A1-RNA interactions can be designed for the treatment of tumors expressing cancer-specific alternatively spliced proteins. Similarly, highly specific inhibitors of the RNA helicase Brr2, which is an essential component of the spliceosome, have been designed for therapeutic purposes (Iwatani-Yoshihara et al., 2017).

Recently, the Massachusetts-based pharmaceutical company Skyhawk Therapeutics invested $100 million in the development of small-molecule compounds capable of correcting misspliced exon skipping related to cancer and neurological diseases. Novartis and Roche have also independently developed two different splicing-modulating compounds for the treatment of SMA: branaplam and risdiplam, respectively. Both molecules enhance exon 7 inclusion to increase the levels of functional SMN protein. Branaplam, also known as LMI070, is an orally available drug that was designed by Novartis using a high-throughput phenotypic screening approach with approximately 1.4 million compounds. Currently, this molecule is in a phase II clinical trial that is expected to be completed in July 2020.

Risdiplam (Ratni et al., 2018) is a brain-penetrant orally administered drug that is currently being evaluated in patients with SMA in four multicenter clinical trials (NCT02913482, NCT02908685, NCT03032172, and NCT03779334). This drug is a splicing modulator that increases exon 7 inclusion in the *SMN2* gene, thereby increasing the levels of SMN protein throughout the organism. Furthermore, this drug is being studied for use in patients of all age ranges with SMA types 1, 2, and 3.

All these examples demonstrate the applicability of small molecules for splicing event modulation and suggest that these molecules are useful as complements and alternatives to oligonucleotides.

#### SMaRT

Spliceosome-mediated RNA trans-splicing is a gene-reprogramming system based on the *trans*-splicing process that can be used for therapeutic applications. The *trans*-splicing methodology is designed to correct aberrant mRNAs by replacing the entire coding sequence upstream or downstream of a target SS. Three different components are involved: the target mRNA, the spliceosome machinery and the pre-*trans*-splicing molecule (PTM), also known as the RNA *trans*-splicing molecule (RTM). The first two components are present in cells, while the third must be provided exogenously. *Trans*-splicing is induced between the exogenous RNA and the endogenous pre-mRNA, producing a chimeric RNA with the wild-type sequence (without mutation/s). To achieve successful correction, the PTM must be correctly designed with the following regions: a binding domain (complementary to the pre-mRNA), a splicing domain (incorporating 5′ and 3′ SS, intronic BPS and Py sequences) and a coding domain (containing the wild-type coding region) (Wally et al., 2012; Berger et al., 2016; Figure 3C).

Depending on the targeted region of the pre-mRNA, SMaRT can be divided into (i) 5′-*trans-*splicing, which targets the 5′ portion; (ii) *3*′*-trans*-splicing, which targets the 3′ portion; and (iii) internal exon replacement (IER), which targets an internal portion of the pre-mRNA (Figure 3C). SMaRT approaches have been used in models of cystic fibrosis (CF) (Song et al., 2009), hemophilia A (Chao et al., 2003), SMA (Coady and Lorson, 2010), retinitis pigmentosa (RP) (Berger et al., 2015), frontotemporal dementia with parkinsonism-17 (FTDP-17) and tauopathies (Rodriguez-Martin et al., 2005, 2009). The results have shown that this technology can successfully reprogram gene expression and offers promising gene therapy applications. However, *trans*-splicing approaches require the use of vectors for the delivery of the PTM into cells. Therefore, selection of a good delivery vector is critical for future treatment approaches based on this technology (Wally et al., 2012). SMaRT offers multiple advantages as a gene therapy tool; however, it needs to be better understood and optimized in order to increase its overall efficiency.

#### Modified snRNAs

Exon-specific U1 snRNAs (ExSpe U1s) are modified U1 snRNAs complementary to intronic regions downstream of the 5′ SS that can be used to eliminate the skipping of some exons caused by different mutations (Figure 3D). ExSpe U1s have been tested in different models and have shown potential for use in therapeutic applications (Dal Mas et al., 2015). In one study, different ExSpe U1s were tested for the treatment of SMA, CD and hemophilia B in *SMN2* exon 7, *CFTR* exon 12 and *F9* exon 5 models, respectively (Fernandez Alanis et al., 2012). Similar approaches have been reported for ATP8B1 deficiency (van der Woerd et al., 2015), FD (Donadon et al., 2018b), Sanfilippo syndrome type C (Matos et al., 2014), Fanconi anemia (Mattioli et al., 2014), RP (Tanner et al., 2009), thalassemia (Gorman et al., 2000), severe coagulation factor VII (FVII) deficiency (hemophilia A, HA) (Pinotti et al., 2008; Donadon et al., 2018a; Balestra et al., 2019b), *CDKL5*-deficiency disorder (CDD) (Balestra et al., 2019a), Seckel syndrome (Scalet et al., 2017), and hereditary tyrosinemia type I (HT1) (Scalet et al., 2018).

Other modified versions of spliceosomal snRNAs have also been tested for their usefulness in restoration of base pairing to the mutated SS. For example, combined treatment with mutation-adapted U1 and U6 snRNAs has been used to correct mutation-induced splice defects in exon 5 of the *BBS1* gene (Schmid et al., 2013); this gene is implicated in Bardet-Biedl syndrome, which causes retinal degeneration and developmental disabilities. Another example of a modified oligonucleotide is U7 snRNA, which participates in histone pre-mRNA maturation by recognizing the sequence of the histone 3′ untranslated region. Changes in the target sequence can be introduced to convert this snRNA into an antisense tool capable of blocking splicing signals and inducing exon skipping or inclusion (Brun et al., 2003). This strategy has been used to design artificial U7 snRNAs to enhance exon 23 skipping of mutated dystrophin in DMD (Brun et al., 2003; Goyenvalle et al., 2004).

The modified snRNA approach is based on engineered variants of small coding genes and has various advantages. Its main advantages are the possible exploitation of virtually any viral vector and the fact that, based on its molecular mechanisms, it does not alter the physiological expression of the target gene.

### Genome Editing Approaches

#### ZFPs

**FIGURE 4:**
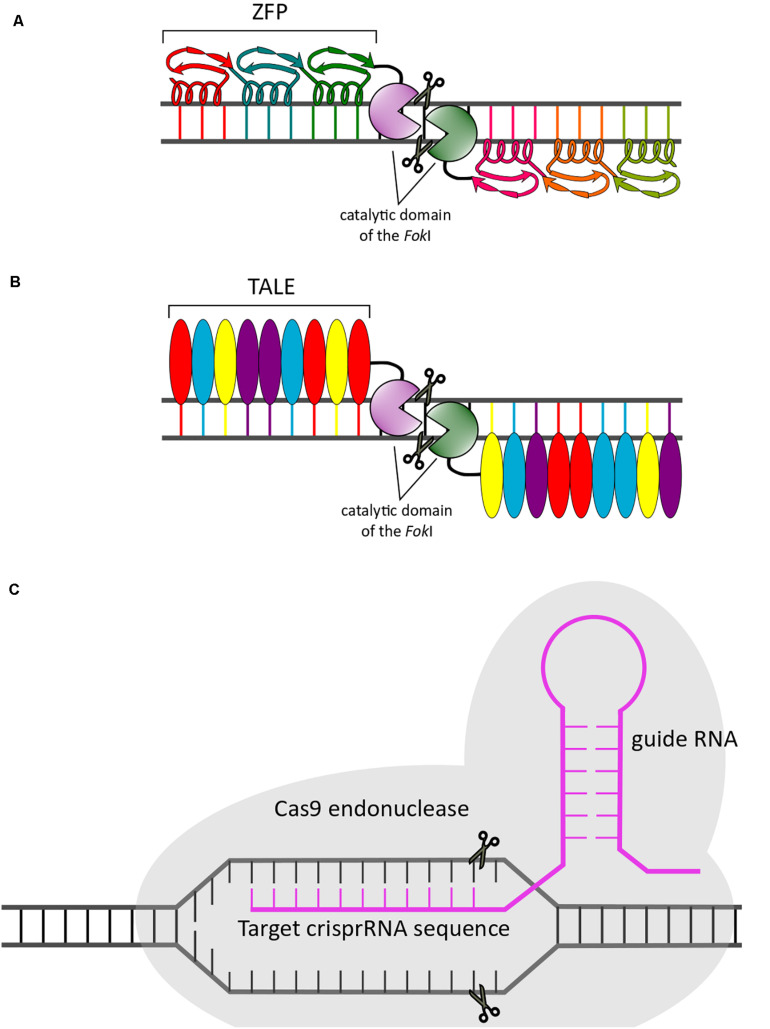
Schematic representation of genome editing approaches. The three gene editing systems described in the main text are illustrated: ZFPs **(A)**, TALENs **(B)**, and CRISPR/Cas9 **(C)**.

Zinc finger proteins are powerful and widely studied tools for efficient establishment of targeted genetic modifications (Cristea et al., 2011). ZFPs designed for therapeutic use consist of ZF arrays in which every ZF element recognizes three bases of a DNA sequence through an α-helix structure (Figure 4A). Examples include zinc finger nucleases (ZFNs) that cleave DNA and zinc finger transcription factors (ZFP-TF) that modulate gene expression (Wild and Tabrizi, 2017).

Zinc finger nucleases were the first endonucleases designed for genome editing. In one such application, the association of ZF domains with the DNA cleavage domain of the restriction protein *Fok*I leads to breakage of a specific region in the DNA sequence (Porteus and Baltimore, 2003). To this end, two different ZFNs must recognize adjacent sequences separated by a spacer sequence where the break will be located. After this step, DNA repair pathways such as nonhomologous end joining (NHEJ) or homologous recombination (HR) with a codelivered exogenous DNA template lead to the establishment of a modified sequence in which the targeted mutation is corrected (Cristea et al., 2011). Unfortunately, the use of ZFNs has some limitations, including high cost, off-target effects due to low specificity, and inappropriate interaction between domains.

Zinc finger nucleases can be used as therapeutic tools to correct genetic mutations associated with splicing-related diseases. The main advantage of ZFNs is that the correction of mutations is permanent; thus, continuous administration is not needed. For instance, Ousterout et al., applied ZFNs for permanent deletion of exon 51 of the dystrophin gene for the treatment of DMD (Ousterout et al., 2015). Other authors have designed ZFPs targeting CAG repeats to decrease the levels of mutant HTT for therapeutic purposes in HD (Garriga-Canut et al., 2012; Zeitler et al., 2019).

The current and completed clinical trials of ZFN therapies (13 in total^1^, accessed in February 2020) focus on three major areas: cancer (1 clinical trial), blood disorders (thalassemia, hemophilia B and sickle-cell disease: 3 clinical trials), infectious diseases (human immunodeficiency virus [HIV]: 7 clinical trials) and orphan diseases (mucopolysaccharidosis: 2 clinical trials).

#### TALENs

These genome editing tools are chimeric nucleases engineered by fusion of the DNA-binding domain of the bacterial protein TALE with the catalytic domain of the restriction endonuclease *Fok*I (Schornack et al., 2006; Figure 4B). Recognition of a specific DNA sequence is performed by the binding domain, which is composed of monomeric tandem repeats of 33–35 conserved amino acids; within this domain is a variable region known as the repeat variable diresidue (RVD) located at residues 12 and 13. The RVD is responsible for binding to a specific nucleotide. Similar to ZFNs, TALENs work as pairs, recognizing sequences separated by 12–25 bp to promote cleavage of the DNA by the *FokI* endonuclease. The advantage of TALENs compared to ZFNs is decreased cytotoxicity due to reduced off-target effects (Sun and Zhao, 2013).

Fang et al. used TALENs *in vivo* for the treatment of β654-thalassemia in a mouse model. The TALENs targeted the mutation site, corrected aberrant β-globin RNA splicing, and ameliorated the β-thalassemia phenotype in β654 mice (Fang et al., 2018). TALENs have also been designed for the treatment of HD (Fink et al., 2016) and DMD (Li et al., 2015). The current trials on TALEN therapies (5 in total^1^, accessed in February 2020) are focused entirely on cancer.

#### CRISPR/Cas9

The development of CRISPR/Cas9 technology is considered one of the most important breakthroughs of the last decade. This nucleic acid immune system was first discovered in bacteria and archaea more than thirty years ago (Ishino et al., 1987; Hermans et al., 1991; Mojica et al., 1993). In 2012, Jennifer Doudna and Emmanuelle Charpentier suggested that this system could be used for RNA-programmable genome editing (Jinek et al., 2012). After that study, Feng Zhang and George Church performed the first *in vitro* studies in eukaryotes demonstrating the genome editing capacity of the CRISPR/Cas9 system in mouse and human cells (Cong et al., 2013; Mali et al., 2013). Since these seminal reports, many researchers have contributed to the molecular understanding, technological development and medical applications of this gene editing system (Lander, 2016).

The CRISPR/Cas9 gene editing system requires administration of two main elements into a cell: (i) a small molecule of RNA (guide RNA [gRNA]) with a sequence complementary to that of the target mutation intended for editing and (ii) an endonuclease CRISPR-associated system (Cas) that allows the cleavage of the specific sequence under the guidance of gRNA binding (Figure 4C). Although many Cas9 orthologs have been investigated, the most widely used is Cas9 from *Streptococcus pyogenes*. In most eukaryotic cells and after cleavage, two repair pathways, NHEJ and homology-directed repair (HDR), are used to correct CRISPR-mediated breaks. The application areas of CRISPR/Cas9 technology go beyond genome editing, and many comprehensive reviews discussing the potential and specific applications of this system in science and medicine have been published (Adli, 2018 and references therein).

Several studies have used CRISPR/Cas9 to correct splicing-related defects. Yuan et al. used a CRISPR-guided cytidine deaminase to successfully correct mutations associated with splicing-related diseases. These researchers used this tool to restore the expression and function of the protein dystrophin in DMD patients (Yuan et al., 2018). Foltz et al. used CRISPR/Cas9 to generate corrected induced pluripotent stem cells (iPSCs) from fibroblasts with a mutation in the PRPF8 splicing factor from patients with RP. These researchers were also able to differentiate each of these clones into retinal pigment epithelial cells with a nearly normal phenotype, highlighting the power and utility of this genome editing tool (Foltz et al., 2018). Dastidar et al. used CRISPR/Cas9 to excise a CTG-repeat expansion of the *DMPK* gene, abnormal length of which leads to sequestration of muscle blind-like (MBLN) splicing factors, and achieved correction efficiencies of up to 90% in myotonic dystrophy type-1 (DM1) iPSCs. These results support the use of this tool in developing new therapies for the treatment of DM1 (Dastidar et al., 2018). CRISPR/Cas9 has also been used to treat the neurodegenerative disease X-linked dystonia-parkinsonism (XDP) by excising the SINE-VNTR-Alu (SVA) retrotransposition in intron 32 of the *TAF1* gene in multiple pluripotent stem cell-derived neuronal lineages. An XDP-specific transcriptional signature with normalized TAF1 expression levels was achieved in these cells (Aneichyk et al., 2018). Kemaladewey et al. demonstrated *in vivo* systemic delivery of an adeno-associated virus (AAV) carrying CRISPR/Cas9 genome editing components in a mouse model of congenital muscular dystrophy type 1A (MDC1A) to correct a pathogenic SS mutation in *LAMA2* pre-mRNA in order to include exon 2. The *LAMA2* gene encodes the α2 chain of the most abundant laminin isoform of the basal lamina (Laminin-211), and restoration of full-length LAMA2 expression by CRISPR/Cas9 improves muscle histopathology and function (Kemaladewi et al., 2017). These observations, together with those described in a follow-up report by the same authors, validate the use of this gene editing technology as a therapeutic strategy for MDC1A (Kemaladewi et al., 2019). Thus far, trials using CRISPR therapies (27 in total^1^, accessed in February 2020) have focused mainly on cancer. Very recently, Stadtmauer et al. reported data from the first phase I clinical trial on cancer immunotherapy combined with CRISPR. The results of the trial demonstrated that it is feasible and safe to apply this technology for cancer immunotherapy (Stadtmauer et al., 2020).

Despite the targeting specificity of Cas9, off-target DNA cleavage activity can occur. In addition, many recent reports have suggested that CRISPR/Cas9 might unintentionally generate alternatively spliced products, large genomic deletions, translocations and inversions; this is a matter of concern that should be further evaluated prior to the clinical application of this technology (Smith et al., 2018).

A modification of the CRISPR/Cas9 system termed the base editing system (BEs) has expanded the arsenal of tools for genome modification. Unlike CRISPR, the BEs can introduce precise base changes without introducing double-strand breaks (DSBs) and, in the case of HDR, without the requirement of a template donor (Hess et al., 2017; Rees and Liu, 2018). Two classes of BEs have been developed: the cytosine BEs (CBEs) (Komor et al., 2016) and the adenine BEs (ABEs) (Gaudelli et al., 2017); these systems include cytidine deaminases and evolved *Escherichia coli* TadA, respectively. Osborn et al. applied the ABEs to an *in vitro* model of primary fibroblasts extracted from recessive dystrophic epidermolysis bullosa (RDEB) patients. Compared with HDR using a donor template, the ABEs efficiently corrected two *COL7A1* mutations with minimal indels (Osborn et al., 2020). Recently, Song et al. also systemically delivered an ABEs and a single gRNA (sgRNA) to edit a point mutation in the *FAH* gene in an adult mouse model of HTI. The results showed improvements in liver histology in ABEs-treated mice, and the correction of the point mutation was confirmed by sequencing, indicating restoration from the diseased to the normal phenotype *in vivo* (Song et al., 2020). The enormous potential of this technique is related to the fact that conversion of A⋅T to G⋅C base pairs in genomic DNA makes it possible to correct almost half of the 32,000 point mutations that cause genetic diseases (Gaudelli et al., 2017).

Some of the strategies presented in this section are feasible treatment options for several diseases. Although the potential of nucleic acids (DNA or RNA) as drugs immediately became obvious decades ago, the actual development of nucleic acid-based medicines has faced major and evident hurdles. For instance, nucleic acids are highly susceptible to degradation by endogenous nucleases. Some of these nucleic acids, such as short oligonucleotides in their native form, have a very short half-life, even before they are filtered out through the kidneys. Large DNA/RNA constructs with highly negative charges cannot cross the vascular endothelium, dense extracellular matrix and cell and nuclear membranes to reach their intracellular DNA, pre-mRNA or mRNA targets. Moreover, off-target effects of many DNA/RNA therapeutic tools can lead to devastating adverse reactions. Finally, some of these tools can be immunogenic. Although chemical modifications can improve pharmacokinetic and pharmacodynamic properties, the ability of these promising therapeutic tools to efficiently deliver DNA/RNA in order to modify sufficient numbers of cells for therapeutic benefits is still the limiting factor for the translation of preclinical models to standard clinical care. This ongoing challenge, which is considered the Achilles heel of gene therapy (Somia and Verma, 2000), is beginning to be overcome through the use of nanotechnologies. These technologies use complexes of nucleic acids or encapsulate the nucleic acids in nonviral vectors, such as liposomes, lipids, and polymeric or inorganic nanoparticles, to enhance safe delivery to the target site. Next, we will focus on the application of nanotechnologies for gene delivery and discuss the advantages and problems associated with nanotechnology-based systems. Addressing the problems will dismantle the barriers facing nucleic acid-based therapeutics.

## Nanomedicine

In recent decades, various vectors and tools have been developed for gene therapy. In addition, the advent of new gene editing therapies, such as CRISPR/Cas9, has sparked investigation into appropriate gene delivery systems, including viral vector and transposon-based vector systems. Nanostructures are nanoscale-sized particles capable of transfecting cells and releasing cargoes such as small molecules, DNA, RNA and peptides to exert pharmacological effects. These nonviral vectors have received considerable attention due to their advantages compared to viral systems, which have been the most common choices for gene delivery. Several good reviews have extensively explained the differences between these types of vectors (Chira et al., 2015; Ramamoorth and Narvekar, 2015; Foldvari et al., 2016). The main advantage of nonviral gene delivery systems is their low immunogenicity, as high immunogenicity can impair viral transduction efficacy. Insertional mutagenesis is also a recognized safety concern associated with viral vectors intended for use in gene therapy (Hacein-Bey-Abina et al., 2003; David and Doherty, 2017), and viral integration is recognized as a common outcome of applications that utilize AAVs for genome editing (Hanlon et al., 2019). The major advantages of viral vectors include strong and prolonged transgene expression, broad cell tropism, and thorough understanding of viral gene function. Compared with viral systems, nanoparticle-mediated nucleic acid delivery systems have the advantages of weak immunogenicity, lack of integration and absence of potential for viral recombination, all of which translate to improved safety (Yin et al., 2014). The development processes and manufacturing capacity for clinical-grade nanoparticles are also advantages of nanoparticle-based methods *versus* viral methods. Nanotechnologies are applicable to a large cohort of patients (Paliwal et al., 2014)., and several nanoparticle-based formulations are already on the market. For example, the siRNA-based drug Onpattro^®^, used for the treatment of the polyneuropathy hATTR in adults via inhibition of the production of the disease-causing protein, is encased in lipid nanoparticles for delivery into the body. However, the transfection efficiency of nanoparticle-based systems is comparatively poor, and poor transfection efficiency is the main limitation for this and other nonviral methods. For this reason, AAV vectors are the most commonly used vectors for nucleic-acid delivery. A good overview of the current status of the clinical translation of viral and nonviral systems for gene therapy has recently been published (Kaemmerer, 2018).

Several formulations based on nanoparticles have demonstrated sustained expression of transported cargoes and long-term achievement of biological effects (Cohen et al., 2000; Shi et al., 2014). Nanoparticles can also achieve successful tissue-specific delivery of biomolecules through different strategies. For example, incorporation of specific antibodies into the nanoparticle surface has enabled effective targeting of nanoparticles to the brain and lung endothelium (Kolhar et al., 2013) for the treatment of many types of cancer, inflammation dysfunction, and infectious disease (Cardoso et al., 2012). Specific chemical components have also been incorporated into nanoparticles to increase delivery of biomolecules to targeted cells. Incorporation of phosphatidylserine (PS), cholesteryl-9-carboxynonanoeate (9-CCN) (Maiseyeu et al., 2010), or folate into targeted nanoparticles (Krzyszton et al., 2017) has been shown to increase uptake by macrophages to help treat atherosclerosis and rheumatoid arthritis.

**FIGURE 5:**
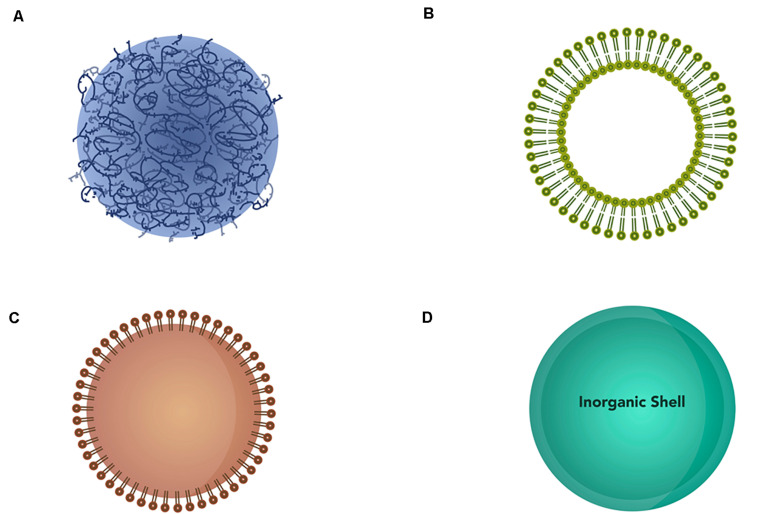
Schematic representation of different types of nanoparticles. **(A)** Polymeric nanoparticles, **(B)** liposomes, **(C)** lipid nanoparticles, and **(D)** inorganic nanoparticles.

Nanoparticles exist in different forms and can be divided into different classes based on their compositions and properties: polymeric nanoparticles, liposomes, lipid nanoparticles, and inorganic nanoparticles (Figure 5).

### Polymeric Nanoparticles

Polymeric nanoparticles are based on a polymeric matrix. The most frequently used polymers are poly-lactide-coglycolide (PLGA), polyhydroxyalkanoates (PHAs) and CDs (CDs) (Zhang et al., 2018). PLGA is a biodegradable and biocompatible polymer formed by units of lactic acid and glycolic acid. This excipient is approved by the FDA and has been extensively used to develop nanoparticles (Zakharova et al., 2017). The grade of the polymer depends on the ratio between lactic acid and polyglycolic acid (PGA), which can affect the final characteristics of the nanoparticle. For example, polymers with higher glycolide contents have shorter deterioration times due to their more hydrophilic and amorphous characteristics. On the other hand, polymers with higher lactic content are more hydrophobic, thus exhibiting longer deterioration times (Schliecker et al., 2003). Frequently, PLGA is mixed with other polymers to improve the characteristics of the resulting nanoparticles. For example, polyethylenimine (PEI) is commonly incorporated to improve the transfection efficiency of nanostructures (Xie et al., 2016; Wang et al., 2018). PEI is a polycationic polymer capable of condensing DNA and RNA into stable nanostructures, primarily via electrostatic interactions. However, several studies have revealed that this polymer is cytotoxic, as elegantly discussed by Hunter more than a decade ago (Hunter, 2006). Thus, the translation of PEI-based nanoparticles to clinical applications is limited. Another polymer frequently mixed with PLGA is polyethylene glycol (PEG) (Zhang et al., 2014). PEG is a nonionic biocompatible polymer coated onto the surfaces of nanoparticles that prevents recognition and destruction of these carriers by the mononuclear phagocyte system (MPS), thereby increasing the plasma half-lives of the nanoparticles (Mustafa et al., 2017). Furthermore, PEGylation of nanoparticles improves their stability by reducing intermolecular aggregation and the accessibility of the target site (Gref et al., 2000). PLGA-based nanoparticles can also be functionalized with ligands such as antibodies and Fab fragments to improve cellular targeting (Kennedy et al., 2018).

Polyhydroxyalkanoates are polyesters produced by microorganisms through, for example, bacterial fermentation of sugars or lipids. Because they are biodegradable and biocompatible polymers, PHAs have been used as bioimplant materials for medical and therapeutic applications for more than thirty years (reviewed in Zhang et al., 2018). Different types of PHAs can be used for nanoparticle formulation. There are 2 types of PHAs with different chain lengths: short-chain-length PHAs (scl-PHAs), which are composed of 3 to 5 carbon atoms, and medium-chain-length PHAs (mcl-PHAs), which are composed of 6 to 16 carbon atoms. There is also a subtype of PHAs that includes copolymers of scl-mcl PHAs of 4 to 12 carbon atoms (Hazer and Steinbuchel, 2007). PHA-based nanoparticles have been used to deliver biomolecules for anticancer (Lu et al., 2011; Fan et al., 2018; Radu et al., 2019) and antibacterial applications (Castro-Mayorga et al., 2014, 2016; Mukheem et al., 2018). Nanocarriers fabricated from PHA−grafted copolymers have also been developed for efficient siRNA delivery (Zhou et al., 2012); these formulations are safe siRNA carriers for gene therapy. A review discussing the use of PHA-based nanovehicles as therapeutic delivery carriers has been recently published (Lin, 2017).

CDs are cyclic oligosaccharides extensively used in pharmaceutical and biomedical applications. These molecules can be divided into 3 groups: α-CDs (6 subunits of glucose), β-CDs (7 subunits of glucose) and γ-CDs (8 subunits of glucose). CDs are biocompatible products approved by the FDA that are currently present in marketed formulations (Jambhekar and Breen, 2016). The cyclic structure of CDs results in a hydrophobic lumen and a hydrophilic surface. This characteristic allows the use of CDs for multiple purposes, such as the vectorization of lipophilic drugs (Fine-Shamir et al., 2017). Furthermore, CDs can penetrate cells and release their cargoes through, for example, pH-dependent mechanisms (Tardy et al., 2016). CD-based nanoparticles have also been used for gene delivery. For example, Zuckerman et al. developed CD nanoparticles containing siRNAs for the treatment of chronic kidney diseases. Additionally, these researchers reported the functionalization of these nanoparticles with mannose or transferrin for enhanced nanoparticle uptake (Zuckerman et al., 2015).

### Liposomes

Liposomes were some of the first nanostructures to be developed for drug delivery. Liposomes were developed in the 1960s and are currently present in marketed formulations such as Doxyl^®^, Myocet^®^ and Caelix^®^. At present, no liposome-based marketed formulations for gene delivery exist. Liposomes are nanoscale particles with a lipid bilayer composition that forms a spherical structure inside an aqueous compartment. In aqueous solution, liposomes form colloidal dispersions. The main components of liposomes are phospholipids, such as phosphatidylcholine (PC), phosphatidylethanolamine (PE), PS, phosphatidylinositol (PI) and phosphatidyl glycerol (PG), and cholesterol, which can be incorporated into the phospholipid membrane to increase liposome stability (Bozzuto and Molinari, 2015). Other excipients can be used to improve the properties of liposomes or to endow them with new characteristics suitable for gene delivery. For example, dioleoylphosphatidylethanolamine (DOPE) is used to produce pH-sensitive liposomes, and the cationic lipids N-[1-(2,3-dioleyloxy)propyl]-N,N,N-trimethylammonium chloride (DOTMA) and N-[1-(2,3-dioleoyloxy)propyl]-N,N,N-trimethylammonium chloride (DOTAP) are used to formulate liposomes with cationic charges to facilitate the incorporation of DNA and RNA. In addition, polymers and carbohydrates, such as PEG and monosialoganglioside (GM1), can be incorporated into nanoparticle formulations to improve their *in vivo* half-lives and stability (Daraee et al., 2016b).

Many published studies have shown the efficacy of liposomes in delivering RNA or DNA into cells. For example, Dorrani et al. developed a liposome formulation with DOTAP and sodium cholate (NaChol) as edge-activators that is capable of efficiently delivering siRNA through skin layers after topical administration, demonstrating that liposomes are good candidates for the treatment of skin diseases such as melanoma (Dorrani et al., 2016). Qiao et al. developed a formulation incorporating mannosylated zwitterionic-based cationic liposomes (man-ZCLs) that increases the endosomal/lysosomal escape of nanostructures to enable administration of a DNA vaccine for HIV, providing a new, safe and effective HIV vaccination method that can be tested in future studies (Qiao et al., 2016). Recently, a four-component liposome formulation with DOTAP, DOPE, PEG, and cholesterol was used to transfect Cas9/sgRNA with high efficacy in order to knock out targeted genes *in vivo* (Hosseini et al., 2019).

### Lipid Nanoparticles

Lipid nanocarriers are some of the most promising nonviral tools for gene therapy. Currently, the only medicine approved by the FDA and European Medicines Agency (EMA) that uses nanostructures to deliver RNA is Onpattro^®^ (mentioned in section “Small Interfering RNAs (siRNAs)”); Onpattro^®^ is a lipid nanoparticle-based drug product that transports patisiran, an siRNA molecule for the treatment of TTR amyloidosis (Rizk and Tuzmen, 2019).

Lipid nanoparticles were developed to address important drawbacks of other lipid-based systems, such as instability and the necessity for use of surfactants and other toxic substances; to increase loading capacity; and to resolve other problems related to manufacturing and scale-up processes (Muller et al., 2000). Lipid nanoparticles can be divided into 2 categories: solid lipid nanoparticles (SLNs) and nanostructured lipid carriers (NLCs) (Gordillo-Galeano and Mora-Huertas, 2018). Both types of nanoparticles use lipid excipients that are biocompatible and biodegradable. These structures are highly attractive for clinical applications due to their simple and inexpensive manufacturing processes that do not require organic solvents and can be easily scaled up; their high stability; and their ability to be administered through different routes, such as the parenteral, pulmonary, oral and topical routes (Uner and Yener, 2007).

Solid lipid nanoparticles have been developed for gene delivery since 2000. The structure of SLNs can help to protect the drug or RNA/DNA against external agents and can enable modified and/or targeted release (Muller et al., 2000). SLNs are manufactured with solid lipid excipients. The most common excipients include stearic acid, cholesterol derivatives (e.g., cholesteryl oleate), glyceryl monostearate, glyceryl behenate, cetylpalmitate, glycolipids, tripalmitine and tristearin. Other essential excipients that are incorporated into these formulations are surfactants and cosurfactants, such as Pluronic^®^ compounds (i.e., Pluronic F68), Poloxamer^®^ compounds (i.e., Poloxamer 188), Brij^®^, Tween 80, and Span 20. Cationic molecules can also be incorporated in formulations to provide a positive surface charge (forming cationic SLNs [cSLNs]) in order to facilitate the formation of SLNplexes with DNA/RNA. The most commonly used cationic excipients are octadecylamine, benzalkonium chloride, cetrimide (DTAB), DOTAP, N,N-di-(β-stearoylethyl)-N,N-dimethyl-ammonium chloride (Esterquat 1 [EQ1]) and stearylamine (de Jesus and Zuhorn, 2015). To improve the efficacy of this type of vector, other excipients, such as protamine (Limeres et al., 2019), can be incorporated into the formulation. There are many examples of the use of SLNs for gene therapy. For example, Apaolaza et al. developed a formulation incorporating hyaluronic acid for transfection of cells with a plasmid containing the RS1 gene. Intravitreal administration of this formulation induced the expression of the protein retinoschisin in photoreceptors of Rs1h-deficient mice, leading to structural improvements in degenerated retinas (Apaolaza et al., 2016). Rassu et al. designed SLNs capable of carrying *BACE1* siRNA to the brain after nasal administration for the treatment of Alzheimer’s disease. These researchers formulated nanoparticles with RVG-9R, a type of cell-penetrating peptide (CPP) that facilitated the transcellular pathway in neuronal cells. Furthermore, the researchers coated the nanoparticles with chitosan, which provided extra protection to the siRNA and increased the mucoadhesiveness of the particles, thereby increasing the residence time in the nasal cavity (Rassu et al., 2017).

Nanostructured lipid carriers were first developed several years after SLNs. In contrast to SLNs, which involve solid lipids, NLCs involve liquid lipids; the use of liquid lipids increases the stability and drug loading capacity of the nanoparticles (Uner and Yener, 2007). The liquid lipids most commonly used to formulate NLCs are oleic acid and caprylic/capric triglycerides. Other lipids used are canola stearin and myristyl myristate. The other excipients are the same as those used for SLNs. A study by Taratula et al. has demonstrated the high loading capacity and gene delivery potential of NLCs. The authors developed a multifunctional NLC-based system containing a drug (paclitaxel or doxorubicin), two different types of siRNA, and a modified synthetic analog of luteinizing hormone-releasing hormone (LHRH) to increase specificity for local targeted delivery to lung tumors (Taratula et al., 2013). Similarly, Chen et al. have shown the capability of NLCs to coencapsulate plasmids and temozolomide, an anticancer drug. Those authors tested the system *in vitro* and *in vivo* for efficient delivery to malignant glioblastoma cells for the treatment of malignant gliomatosis cerebri (Chen et al., 2016).

### Inorganic Nanoparticles

Inorganic nanoparticles include nanostructures that are manufactured using inorganic materials, such as gold, silicon and iron oxide; carbon materials; layered double hydroxide (LDH); or calcium phosphate (Xu et al., 2006). The easy surface functionalization, good target delivery and controlled release of these nanoparticles are their main advantages. Some of the most widely used inorganic nanostructures are gold nanoparticles (AuNPs). This type of nanoparticle is used in the biomedical field for different applications, such as biodetection, biodiagnostics, and bioelectronic or therapeutic agent development. Among the advantages of these nanoparticles are their size- and structure-dependent visual and electronic characteristics and high surface/volume ratios and the capability to functionalize their surfaces due to their high affinities for different functional groups (Giljohann et al., 2010; Daraee et al., 2016a). One example of an AuNP-based gene delivery system was recently developed by Jia et al. (2017). The authors surface-conjugated AuNPs with thiol-modified antago-miR155, an RNA antagonist to a potent promoter of proinflammatory type 1 macrophage polarization (miR155) that plays an important role in diabetic cardiomyopathy. *In vivo* administration of the AuNP complex resulted in the incorporation of nucleic acids into macrophages via phagocytosis and led to reduced inflammation, reduced apoptosis and restoration of cardiac function. AuNPs have also been used to deliver Cas9 ribonucleoprotein and donor DNA *in vitro* and *in vivo* and to correct the DNA mutation in the dystrophin gene that causes DMD (Lee et al., 2017).

Oxide nanoparticles can be classified into two important groups: silicon oxide nanoparticles and iron nanoparticles (Xu et al., 2006). Mesoporous silica nanoparticles (MSNs) have been extensively investigated with regard to gene delivery. Indeed, RNA can be loaded onto the surfaces of small pore-sized MSNs, thereby enabling RNA delivery into cells. Furthermore, RNA and drugs can be loaded onto the same large pore-sized MSNs; thus, large pore-sized MSNs have the capacity to deliver two therapeutic agents at the same time. Sun et al. developed core-shell hierarchical MSNs (H-MSNs); doxorubicin can be loaded into the core mesopores, and siRNAs that downregulate the expression of P-gp to reverse multidrug resistance (MDR) can be loaded into the shell mesopores. The specific release of siRNA into the microtumor environment enables inhibition of MDR, and the subsequent release of doxorubicin enhances this effect (Sun et al., 2017).

Iron nanoparticles can be made of different materials, such as magnetite (Fe_3_O_4_) and maghemite (Fe_2_O_3_). The intrinsic magnetic properties of these nanoparticles can be used for gene or drug delivery. For example, an external magnetic field can be used to guide the nanoparticles to a specific zone of the body for drug release. Furthermore, a magnetic field can be applied to a cell culture dish to enhance cell transfection by magnetic nanoparticles in a procedure known as magnetofection (Estelrich et al., 2015). Despite the difficulties encountered in translating the magnetofection technique to clinical applications, advanced studies have demonstrated that iron nanoparticles can be applied for *ex vivo* delivery of chemically modified RNA (cmRNA), opening the door to continuing studies on gene therapy applications (Badieyan et al., 2017).

## Concluding Remarks

In 1993, the Nobel Prize in Physiology and Medicine was awarded to Phillip Sharp and Richard Roberts for their discovery of adenoviral RNA splicing (Berget et al., 1977; Chow et al., 1977). This discovery had notable consequences for elucidation of gene expression regulation and the evolution of eukaryotic cells. More than forty years after this seminal discovery, we have a deep understanding of the molecular mechanisms that control this important regulatory process, and we have recently begun to unravel the molecular links that connect faulty splicing with many human disorders. This knowledge has enabled the design of innovative therapeutic strategies intended to correct splicing defects. Many tools based on nucleic acid gene repair have been tested with positive results, and many more tools warrant further development. The field is moving notably quickly, but we have attempted to provide a general overview of the main developments. However, as important as it is to decipher the mechanisms that govern the connections between missplicing and pathologies and to apply these findings in the clinic, research on the safe transport of therapeutic biomolecules into cells and to their targets is equally important. Further development, characterization and testing of engineered technologies for targeted delivery and controlled release of DNA and RNA directly into cells with clinical applications are needed, as the demand for innovative nucleic acid delivery systems continues to grow. Nanoparticles possess considerable potential for use in the controlled delivery of therapeutic agents to specific target sites for splicing-based treatments. Conducting related research is a challenging task, as basic scientists must interact and collaborate with nanotechnology experts. Funding opportunities should emphasize such collaboration as a way forward for grant support.

## Author Contributions

All authors contributed to the writing and revision of the manuscript and approved the submitted version.

## Conflict of Interest

The authors declare that the research was conducted in the absence of any commercial or financial relationships that could be construed as a potential conflict of interest.

## Footnotes

1 https://clinicaltrials.gov

## References

[B1] Adli M. (2018). The CRISPR tool kit for genome editing and beyond. *Nat. Commun.* 9:1911. 10.1038/s41467-018-04252-2 PMC5953931 29765029

[B2] Aird D. Teng T. Huang C. L. Pazolli E. Banka D. Cheung-Ong K. (2019). Sensitivity to splicing modulation of BCL2 family genes defines cancer therapeutic strategies for splicing modulators. *Nat. Commun.* 10:137. 10.1038/s41467-018-08150-5 PMC6329755 30635584

[B3] Alves C. A. Benito-Vicente A. Medeiros A. M. Reeves K. Martin C. Bourbon M. (2018). Further evidence of novel APOB mutations as a cause of familial hypercholesterolaemia. *Atherosclerosis.* 277 448–456. 10.1016/j.atherosclerosis.2018.06.819 30270084

[B4] Aneichyk T. Hendriks W. T. Yadav R. Shin D. Gao D. Vaine C. A. (2018). Dissecting the causal mechanism of X-linked dystonia-parkinsonism by integrating genome and transcriptome assembly. *Cell.* 172 897.e821–909.e281. 29474918 10.1016/j.cell.2018.02.011 PMC5831509

[B5] Anna A. Monika G. (2018). Splicing mutations in human genetic disorders: examples, detection, and confirmation. *J. Appl. Genet.* 59 253–268. 10.1007/s13353-018-0444-7 29680930 PMC6060985

[B6] Apaolaza P. S. Del Pozo-Rodriguez A. Solinis M. A. Rodriguez J. M. Friedrich U. Torrecilla J. (2016). Structural recovery of the retina in a retinoschisin-deficient mouse after gene replacement therapy by solid lipid nanoparticles. *Biomaterials* 90 40–49. 10.1016/j.biomaterials.2016.03.004 26986855

[B7] Arnold E. S. Ling S. C. Huelga S. C. Lagier-Tourenne C. Polymenidou M. Ditsworth D. (2013). ALS-linked TDP-43 mutations produce aberrant RNA splicing and adult-onset motor neuron disease without aggregation or loss of nuclear TDP-43. *Proc. Natl. Acad. Sci. U.S.A.* 110 E736–E745. 23382207 10.1073/pnas.1222809110 PMC3581922

[B8] Axelrod F. B. Liebes L. Gold-Von Simson G. Mendoza S. Mull J. Leyne M. (2011). Kinetin improves IKBKAP mRNA splicing in patients with familial dysautonomia. *Pediatr. Res.* 70 480–483. 10.1203/pdr.0b013e31822e1825 21775922 PMC3189334

[B9] Aznarez I. Chan E. M. Zielenski J. Blencowe B. J. Tsui L. C. (2003). Characterization of disease-associated mutations affecting an exonic splicing enhancer and two cryptic splice sites in exon 13 of the cystic fibrosis transmembrane conductance regulator gene. *Hum. Mol. Genet.* 12 2031–2040. 10.1093/hmg/ddg215 12913074

[B10] Badieyan Z. S. Pasewald T. Mykhaylyk O. Rudolph C. Plank C. (2017). Efficient ex vivo delivery of chemically modified messenger RNA using lipofection and magnetofection. *Biochem. Biophys. Res. Commun.* 482 796–801. 10.1016/j.bbrc.2016.11.113 27888105

[B11] Bailey P. Chang D. K. Nones K. Johns A. L. Patch A. M. Gingras M. C. (2016). Genomic analyses identify molecular subtypes of pancreatic cancer. *Nature.* 531 47–52. 26909576 10.1038/nature16965

[B12] Baker M. S. Ahn S. B. Mohamedali A. Islam M. T. Cantor D. Verhaert P. D. (2017). Accelerating the search for the missing proteins in the human proteome. *Nat. Commun.* 8:14271. 10.1038/ncomms14271 PMC5286205 28117396

[B13] Baker N. L. Mörgelin M. Peat R. Goemans N. North K. N. Bateman J. F. (2005). Dominant collagen VI mutations are a common cause of Ullrich congenital muscular dystrophy. *Hum. Mol. Genet.* 14 279–293. 10.1093/hmg/ddi025 15563506

[B14] Balestra D. Giorgio D. Bizzotto M. Fazzari M. Ben Zeev B. Pinotti M. (2019a). Splicing mutations impairing CDKL5 expression and activity can be efficiently rescued by U1snRNA-based therapy. *Int. J. Mol. Sci.* 20:4130. 10.3390/ijms20174130 31450582 PMC6747535

[B15] Balestra D. Maestri I. Branchini A. Ferrarese M. Bernardi F. Pinotti M. (2019b). An altered splicing registry explains the differential ExSpeU1-mediated rescue of splicing mutations causing haemophilia A. *Front. Genet.* 10:974. 10.3389/fgene.2019.00974 31649737 PMC6796300

[B16] Barbaux S. Niaudet P. Gubler M. C. Grünfeld J. P. Jaubert F. Kuttenn F. (1997). Donor splice-site mutations in WT1 are responsible for Frasier syndrome. *Nat. Genet.* 17 467–470. 10.1038/ng1297-467 9398852

[B17] Berger A. Lorain S. Josephine C. Desrosiers M. Peccate C. Voit T. (2015). Repair of rhodopsin mRNA by spliceosome-mediated RNA trans-splicing: a new approach for autosomal dominant retinitis pigmentosa. *Mol. Ther.* 23 918–930. 10.1038/mt.2015.11 25619725 PMC4427870

[B18] Berger A. Maire S. Gaillard M. C. Sahel J. A. Hantraye P. Bemelmans A. P. (2016). mRNA trans-splicing in gene therapy for genetic diseases. *Wiley Interdiscipl. Rev. RNA* 7 487–498. 10.1002/wrna.1347 27018401 PMC5071737

[B19] Berget S. M. Moore C. Sharp P. A. (1977). Spliced segments at the 5’ terminus of adenovirus 2 late mRNA. *Proc. Natl. Acad. Sci. U.S.A.* 74 3171–3175. 10.1073/pnas.74.8.3171 269380 PMC431482

[B20] Bolduc V. Zou Y. Ko D. Bonnemann C. G. (2014). siRNA-mediated allele-specific silencing of a COL6A3 mutation in a cellular model of dominant ullrich muscular dystrophy. *Mol. Ther. Nucleic Acids* 3:e147. 10.1038/mtna.2013.74 24518369 PMC3950771

[B21] Bourbon M. Duarte M. A. Alves A. C. Medeiros A. M. Marques L. Soutar A. K. (2009). Genetic diagnosis of familial hypercholesterolaemia: the importance of functional analysis of potential splice-site mutations. *J. Med. Genet.* 46 352–357. 10.1136/jmg.2007.057000 19411563

[B22] Bowen D. J. (2002). Haemophilia A and haemophilia B: molecular insights. *Mol. Pathol.* 55 127–144. 10.1136/mp.55.2.127 11950963 PMC1187163

[B23] Bozzuto G. Molinari A. (2015). Liposomes as nanomedical devices. *Int. J. Nanomed.* 10 975–999. 10.2147/IJN.S68861 PMC4324542 25678787

[B24] Brooks A. N. Choi P. S. de Waal L. Sharifnia T. Imielinski M. Saksena G. (2014). A pan-cancer analysis of transcriptome changes associated with somatic mutations in U2AF1 reveals commonly altered splicing events. *PLoS ONE* 9:e87361. 10.1371/journal.pone.0087361 24498085 PMC3909098

[B25] Brosseau J. P. Lucier J. F. Lamarche A. A. Shkreta L. Gendron D. Lapointe E. (2014). Redirecting splicing with bifunctional oligonucleotides. *Nucleic Acids Res.* 42 e40. 10.1093/nar/gkt1287 24375754 PMC3973305

[B26] Brun C. Suter D. Pauli C. Dunant P. Lochmuller H. Burgunder J. M. (2003). U7 snRNAs induce correction of mutated dystrophin pre-mRNA by exon skipping. *Cell Mol. Life Sci.* 60 557–566. 10.1007/s000180300047 12737315 PMC11138867

[B27] Cacace R. Sleegers K. Van Broeckhoven C. (2016). Molecular genetics of early-onset Alzheimer disease revisited. *Alzheimers Dement*. 12 733–748. 10.1016/j.jalz.2016.01.012 27016693

[B28] Camacho Vanegas O. Bertini E. Zhang R. Z. Petrini S. Minosse C. Sabatelli P. (2001). Ullrich scleroatonic muscular dystrophy is caused by recessive mutations in collagen type VI. *Proc. Natl. Acad. Sci. U.S.A*. 98 7516–7521. 10.1073/pnas.121027598 11381124 PMC34700

[B29] Campbell M. A. Wengel J. (2011). Locked vs. unlocked nucleic acids (LNA vs. UNA): contrasting structures work towards common therapeutic goals. *Chem. Soc. Rev.* 40 5680–5689. 21556437 10.1039/c1cs15048k

[B30] Carabet L. A. Leblanc E. Lallous N. Morin H. Ghaidi F. Lee J. (2019). Computer-aided discovery of small molecules targeting the RNA splicing activity of hnRNP A1 in castration-resistant prostate cancer. *Molecules* 24 763. 10.3390/molecules24040763 30791548 PMC6413181

[B31] Cardoso M. M. Peca I. N. Roque A. C. (2012). Antibody-conjugated nanoparticles for therapeutic applications. *Curr. Med. Chem* 19 3103–3127. 10.2174/092986712800784667 22612698

[B32] Castro-Mayorga J. L. Fabra M. J. Cabedo L. Lagaron J. M. (2016). On the Use of the electrospinning coating technique to produce antimicrobial polyhydroxyalkanoate materials containing in situ-stabilized silver nanoparticles. *Nanomaterials (Basel)* 7:4. 10.3390/nano7010004 28336838 PMC5295194

[B33] Castro-Mayorga J. L. Martinez-Abad A. Fabra M. J. Olivera C. Reis M. Lagaron J. M. (2014). Stabilization of antimicrobial silver nanoparticles by a polyhydroxyalkanoate obtained from mixed bacterial culture. *Int. J. Biol. Macromol.* 71 103–110. 10.1016/j.ijbiomac.2014.06.059 25043131

[B34] Chabot B. Shkreta L. (2016). Defective control of pre-messenger RNA splicing in human disease. *J. Cell. Biol.* 212 13–27. 10.1083/jcb.201510032 26728853 PMC4700483

[B35] Chao H. Mansfield S. G. Bartel R. C. Hiriyanna S. Mitchell L. G. Garcia-Blanco M. A. (2003). Phenotype correction of hemophilia A mice by spliceosome-mediated RNA trans-splicing. *Nat. Med.* 9 1015–1019. 10.1038/nm900 12847523

[B36] Chen G. Ning B. Shi T. (2019). Single-cell RNA-Seq technologies and related computational data analysis. *Front. Genet.* 10:317. 10.3389/fgene.2019.00317 31024627 PMC6460256

[B37] Chen Z. Lai X. Song S. Zhu X. Zhu J. (2016). Nanostructured lipid carriers based temozolomide and gene co-encapsulated nanomedicine for gliomatosis cerebri combination therapy. *Drug Deliv.* 23 1369–1373. 26017099 10.3109/10717544.2015.1038857

[B38] Chira S. Jackson C. S. Oprea I. Ozturk F. Pepper M. S. Diaconu I. (2015). Progresses towards safe and efficient gene therapy vectors. *Oncotarget.* 6 30675–30703. 10.18632/oncotarget.5169 26362400 PMC4741561

[B39] Chow L. T. Gelinas R. E. Broker T. R. Roberts R. J. (1977). An amazing sequence arrangement at the 5’ ends of adenovirus 2 messenger RNA. *Cell. Sep* 12 1–8. 10.1016/0092-8674(77)90180-5 902310

[B40] Cieply B. Carstens R. P. (2015). Functional roles of alternative splicing factors in human disease. *Wiley Interdiscipl. Rev. RNA* 311–326. 10.1002/wrna.1276 25630614 PMC4671264

[B41] Cladaras C. Hadzopoulou-Cladaras M. Felberg B. K. Pavlakis G. Zannis V. I. (1987). The molecular basis of a familial ApoE deficiency: an acceptor splice site mutation in the third intron of the deficient apoE gene. *J. Biol. Chem.* 262 2310–2315. 3029073

[B42] Coady T. H. Lorson C. L. (2010). Trans-splicing-mediated improvement in a severe mouse model of spinal muscular atrophy. *J. Neurosci.* 30 126–130. 10.1523/jneurosci.4489-09.2010 20053895 PMC2836862

[B43] Cohen H. Levy R. J. Gao J. Fishbein I. Kousaev V. Sosnowski S. (2000). Sustained delivery and expression of DNA encapsulated in polymeric nanoparticles. *Gene Ther.* 7 1896–1905. 10.1038/sj.gt.3301318 11127577

[B44] Cong L. Ran F. A. Cox D. Lin S. Barretto R. Habib N. (2013). Multiplex genome engineering using CRISPR/Cas systems. *Science* 339 819–823. 23287718 10.1126/science.1231143 PMC3795411

[B45] Coutinho M. F. Lacerda L. Prata M. J. Ribeiro H. Lopes L. Ferreira C. (2008). Molecular characterization of portuguese patients with mucopolysaccharidosis IIIC: two novel mutations in the HGSNAT gene. *Clin. Genet.* 74 194–195. 10.1111/j.1399-0004.2008.01040.x 18518886

[B46] Cristea S. Gregory P. D. Urnov F. D. Cost G. J. (2011). Dissection of splicing regulation at an endogenous locus by zinc-finger nuclease-mediated gene editing. *PLoS ONE* 6:e16961. 10.1371/journal.pone.0016961 21347446 PMC3035666

[B47] Cuyvers E. Sleegers K. (2016). Genetic variations underlying Alzheimer’s disease: evidence from genome-wide association studies and beyond. *Lancet Neurol.* 15 857–868. 10.1016/s1474-4422(16)00127-7 27302364

[B48] Daguenet E. Dujardin G. Valcarcel J. (2015). The pathogenicity of splicing defects: mechanistic insights into pre-mRNA processing inform novel therapeutic approaches. *EMBO Rep.* 16 1640–1655. 10.15252/embr.201541116 26566663 PMC4693517

[B49] Daiger S. P. Bowne S. J. Sullivan L. S. (2014). Genes and mutations causing autosomal dominant retinitis pigmentosa. *Cold Spring Harb. Perpect Med.* 5:a017129. 10.1101/cshperspect.a017129 25304133 PMC4588133

[B50] Dal Mas A. Rogalska M. E. Bussani E. Pagani F. (2015). Improvement of SMN2 pre-mRNA processing mediated by exon-specific U1 small nuclear RNA. *Am. J. Hum. Genet.* 96 93–103. 10.1016/j.ajhg.2014.12.009 25557785 PMC4289686

[B51] Dana H. Chalbatani G. M. Mahmoodzadeh H. Karimloo R. Rezaiean O. Moradzadeh A. (2017). Molecular mechanisms and biological functions of siRNA. *Int. J. Biomed. Sci.* 13 48–57. 28824341 PMC5542916

[B52] Daraee H. Eatemadi A. Abbasi E. Fekri Aval S. Kouhi M. Akbarzadeh A. (2016a). Application of gold nanoparticles in biomedical and drug delivery. *Artif. Cells Nanomed. Biotechnol.* 44 410–422. 25229833 10.3109/21691401.2014.955107

[B53] Daraee H. Eatemadi A. Kouhi M. Alimirzalu S. Akbarzadeh A. (2016b). Application of liposomes in medicine and drug delivery. *Artif Cells Nanomed. Biotechnol.* 44 381–391. 25222036 10.3109/21691401.2014.953633

[B54] Dastidar S. Ardui S. Singh K. Majumdar D. Nair N. Fu Y. (2018). Efficient CRISPR/Cas9-mediated editing of trinucleotide repeat expansion in myotonic dystrophy patient-derived iPS and myogenic cells. *Nucleic Acids Res.* 46 8275–8298. 10.1093/nar/gky548 29947794 PMC6144820

[B55] David R. M. Doherty A. T. (2017). Viral vectors: the road to reducing genotoxicity. *Toxicol Sci.* 155 315–325. 10.1093/toxsci/kfw220 27803388

[B56] de Jesus M. B. Zuhorn I. S. (2015). Solid lipid nanoparticles as nucleic acid delivery system: properties and molecular mechanisms. *J. Control. Release* 201 1–13. 10.1016/j.jconrel.2015.01.010 25578828

[B57] de la Mata M. Alonso C. R. Kadener S. Fededa J. P. Blaustein M. Pelisch F. (2003). A slow RNA polymerase II affects alternative splicing in vivo. *Mol Cell.* 12 525–532. 10.1016/j.molcel.2003.08.001 14536091

[B58] den Hollander A. I. Koenekoop R. K. Yzer S. Lopez I. Arends M. L. Voesenek K. E. J. (2006). Mutations in the CEP290 (NPHP6) gene are a frequent cause of leber congenital amaurosis. *Am. J. Hum. Genet.* 79 556–561. 10.1086/507318 16909394 PMC1559533

[B59] Dessein A. F. Fontaine M. Andresen B. S. Gregersen N. Brivet M. Rabier D. (2010). A novel mutation of the ACADM gene (c.*145C*> G) associated with the common c.985A > G mutation on the other ACADM allele causes mild MCAD deficiency: a case report. *Orphanet J. Rare Dis*. 5 26. 10.1186/1750-1172-5-26 20923556 PMC2967532

[B60] Dewaele M. Tabaglio T. Willekens K. Bezzi M. Teo S. X. Low D. H. (2016). Antisense oligonucleotide-mediated MDM4 exon 6 skipping impairs tumor growth. *J. Clin. Investig.* 126 68–84. 26595814 10.1172/JCI82534 PMC4701541

[B61] Dickson A. Osman E. Lorson C. L. (2008). A negatively acting bifunctional RNA increases survival motor neuron both in vitro and in vivo. *Hum. Gene Ther.* 19 1307–1315. 10.1089/hum.2008.067 19848583 PMC2940461

[B62] Disset A. Bourgeois C. F. Benmalek N. Claustres M. Stevenin J. Tuffery-Giraud S. (2006). An exon skipping-associated nonsense mutation in the dystrophin gene uncovers a complex interplay between multiple antagonistic splicing elements. *Hum. Mol. Genet.* 15 999–1013. 10.1093/hmg/ddl015 16461336

[B63] Dominski Z. Kole R. (1993). Restoration of correct splicing in thalassemic pre-mRNA by antisense oligonucleotides. *Proc. Natl. Acad. Sci. U.S.A.* 90 8673–8677. 10.1073/pnas.90.18.8673 8378346 PMC47420

[B64] Donadon I. McVey J. H. Garagiola I. Branchini A. Mortarino M. Peyvandi F. (2018a). Clustered F8 missense mutations cause hemophilia A by combined alteration of splicing and protein biosynthesis and activity. *Haematologica* 103 344–350. 10.3324/haematol.2017.178327 29170251 PMC5792279

[B65] Donadon I. Pinotti M. Rajkowska K. Pianigiani G. Barbon E. Morini E. (2018b). Exon-specific U1 snRNAs improve ELP1 exon 20 definition and rescue ELP1 protein expression in a familial dysautonomia mouse model. *Hum. Mol. Genet.* 27 2466–2476. 10.1093/hmg/ddy151 29701768 PMC6030917

[B66] Dorrani M. Garbuzenko O. B. Minko T. Michniak-Kohn B. (2016). Development of edge-activated liposomes for siRNA delivery to human basal epidermis for melanoma therapy. *J. Control. Release* 228 150–158. 10.1016/j.jconrel.2016.03.010 26965957

[B67] Dujardin G. Lafaille C. de la Mata M. Marasco L. E. Munoz M. J. Le Jossic-Corcos C. (2014). How slow RNA polymerase II elongation favors alternative exon skipping. *Mol. Cell* 54 683–690. 10.1016/j.molcel.2014.03.044 24793692

[B68] Eckstein F. (2014). Phosphorothioates, essential components of therapeutic oligonucleotides. *Nucleic Therap.* 24 374–387. 10.1089/nat.2014.0506 25353652

[B69] Elbashir S. M. Harborth J. Lendeckel W. Yalcin A. Weber K. Tuschl T. (2001). Duplexes of 21-nucleotide RNAs mediate RNA interference in cultured mammalian cells. *Nature* 411 494–498. 10.1038/35078107 11373684

[B70] Estelrich J. Escribano E. Queralt J. Busquets M. A. (2015). Iron oxide nanoparticles for magnetically-guided and magnetically-responsive drug delivery. *Int. J. Mol. Sci.* 16 8070–8101. 10.3390/ijms16048070 25867479 PMC4425068

[B71] Fadista J. Vikman P. Laakso E. O. Mollet I. G. Esguerra J. L. Taneera J. (2014). Global genomic and transcriptomic analysis of human pancreatic islets reveals novel genes influencing glucose metabolism. *Proc. Natl. Acad. Sci. U.S.A.* 111 13924–13929. 10.1073/pnas.1402665111 25201977 PMC4183326

[B72] Fan F. Wang L. Ouyang Z. Wen Y. Lu X. (2018). Development and optimization of a tumor targeting system based on microbial synthesized PHA biopolymers and PhaP mediated functional modification. *Appl. Microbiol. Biotechnol.* 102 3229–3241. 10.1007/s00253-018-8790-2 29497797

[B73] Fang Y. Cheng Y. Lu D. Gong X. Yang G. Gong Z. (2018). Treatment of beta(654) -thalassaemia by TALENs in a mouse model. *Cell Prolif.* 51 e12491. 10.1111/cpr.12491 30070404 PMC6528953

[B74] Fernandez A. Josa S. Montoliu L. (2017). A history of genome editing in mammals. *Mamm Genome* 28 237–246. 10.1007/s00335-017-9699-2 28589393

[B75] Fernandez Alanis E. Pinotti M. Dal Mas A. Balestra D. Cavallari N. Rogalska M. E. (2012). An exon-specific U1 small nuclear RNA (snRNA) strategy to correct splicing defects. *Hum. Mol. Genet.* 21 2389–2398. 10.1093/hmg/dds045 22362925 PMC3349419

[B76] Ferrari S. Di Iorio E. Barbaro V. Ponzin D. Sorrentino F. S. Parmeggiani F. (2011). Retinitis pigmentosa: genes and disease mechanisms. *Curr. Genomics* 12 238–249. 10.2174/138920211795860107 22131869 PMC3131731

[B77] Fine J. D. Hintner H. (2009). *Life with Epidermolysis Bullosa (EB): Etiology, Diagnosis, Multidisciplinary Care and Therapy.* Berlin: Springer.

[B78] Fine-Shamir N. Beig A. Zur M. Lindley D. Miller J. M. Dahan A. (2017). Toward successful cyclodextrin based solubility-enabling formulations for oral delivery of lipophilic drugs: solubility-permeability trade-off, biorelevant dissolution, and the unstirred water layer. *Mol. Pharm.* 14 2138–2146. 10.1021/acs.molpharmaceut.7b00275 28505451

[B79] Fink K. D. Deng P. Gutierrez J. Anderson J. S. Torrest A. Komarla A. (2016). Allele-specific reduction of the mutant huntingtin allele using transcription activator-like effectors in human huntington’s disease fibroblasts. *Cell Transplant.* 25 677–686. 10.3727/096368916x690863 26850319 PMC6476541

[B80] Fire A. Xu S. Montgomery M. K. Kostas S. A. Driver S. E. Mello C. C. (1998). Potent and specific genetic interference by double-stranded RNA in *Caenorhabditis elegans*. *Nature* 391 806–811. 10.1038/35888 9486653

[B81] Foldvari M. Chen D. W. Nafissi N. Calderon D. Narsineni L. Rafiee A. (2016). Non-viral gene therapy: gains and challenges of non-invasive administration methods. *J. Control. Release* 240 165–190. 10.1016/j.jconrel.2015.12.012 26686079

[B82] Foltz L. P. Howden S. E. Thomson J. A. Clegg D. O. (2018). Functional assessment of patient-derived retinal pigment epithelial cells edited by CRISPR/Cas9. *Int. J. Mol. Sci.* 19:4127. 10.3390/ijms19124127 30572641 PMC6321630

[B83] Fong N. Kim H. Zhou Y. Ji X. Qiu J. Saldi T. (2014). Pre-mRNA splicing is facilitated by an optimal RNA polymerase II elongation rate. *Genes Dev.* 28 2663–2676. 10.1101/gad.252106.114 25452276 PMC4248296

[B84] Furney S. J. Pedersen M. Gentien D. Dumont A. G. Rapinat A. Desjardins L. (2013). SF3B1 mutations are associated with alternative splicing in uveal melanoma. *Cancer Discov.* 3 1122–1129. 10.1158/2159-8290.cd-13-0330 23861464 PMC5321577

[B85] Garriga-Canut M. Agustin-Pavon C. Herrmann F. Sanchez A. Dierssen M. Fillat C. (2012). Synthetic zinc finger repressors reduce mutant huntingtin expression in the brain of R6/2 mice. *Proc. Natl. Acad. Sci. U.S.A.* 109 E3136–E3145. 23054839 10.1073/pnas.1206506109 PMC3494930

[B86] Gaudelli N. M. Komor A. C. Rees H. A. Packer M. S. Badran A. H. Bryson D. I. (2017). Programmable base editing of A^∗^T to G^∗^C in genomic DNA without DNA cleavage. *Nature* 551 464–471. 10.1038/nature24644 29160308 PMC5726555

[B87] Geary R. S. Watanabe T. A. Truong L. Freier S. Lesnik E. A. Sioufi N. B. (2001). Pharmacokinetic properties of 2’-O-(2-methoxyethyl)-modified oligonucleotide analogs in rats. *J. Pharmacol. Exp. Therap.* 296 890–897. 11181921

[B88] Giardine B. van Baal S. Kaimakis P. Riemer C. Miller W. Samara M. (2007). HbVar database of human hemoglobin variants and thalassemia mutations: 2007 update. *Hum. Mutat.* 28 206. 10.1002/humu.9479 17221864

[B89] Giljohann D. A. Seferos D. S. Daniel W. L. Massich M. D. Patel P. C. Mirkin C. A. (2010). Gold nanoparticles for biology and medicine. *Angew. Chem. Int. Ed. Engl.* 49 3280–3294. 20401880 10.1002/anie.200904359 PMC3930332

[B90] Gordillo-Galeano A. Mora-Huertas C. E. (2018). Solid lipid nanoparticles and nanostructured lipid carriers: a review emphasizing on particle structure and drug release. *Eur. J. Pharm. Biopharm.* 133 285–308. 10.1016/j.ejpb.2018.10.017 30463794

[B91] Gorman L. Mercatante D. R. Kole R. (2000). Restoration of correct splicing of thalassemic beta-globin pre-mRNA by modified U1 snRNAs. *J. Biol. Chem.* 275 35914–35919. 10.1074/jbc.m006259200 10969081

[B92] Goyenvalle A. Vulin A. Fougerousse F. Leturcq F. Kaplan J. C. Garcia L. (2004). Rescue of dystrophic muscle through U7 snRNA-mediated exon skipping. *Science* 306 1796–1799. 10.1126/science.1104297 15528407

[B93] Gref R. Luck M. Quellec P. Marchand M. Dellacherie E. Harnisch S. (2000). ‘Stealth’ corona-core nanoparticles surface modified by polyethylene glycol (PEG): influences of the corona (PEG chain length and surface density) and of the core composition on phagocytic uptake and plasma protein adsorption. *Colloids Surf. B Biointerfaces* 18 301–313. 10.1016/s0927-7765(99)00156-3 10915952

[B94] Habara Y. Takeshima Y. Awano H. Okizuka Y. Zhang Z. Saiki K. (2009). In vitro splicing analysis showed that availability of a cryptic splice site is not a determinant for alternative splicing patterns caused by (1G–A mutations in introns of the dystrophin gene. *J. Med. Genet*. 46 542–547. 10.1136/jmg.2008.061259 19001018

[B95] Hacein-Bey-Abina S. Von Kalle C. Schmidt M. McCormack M. P. Wulffraat N. Leboulch P. (2003). LMO2-associated clonal T cell proliferation in two patients after gene therapy for SCID-X1. *Science* 302 415–419. 10.1126/science.1088547 14564000

[B96] Hanlon K. S. Kleinstiver B. P. Garcia S. P. Zaborowski M. P. Volak A. Spirig S. E. (2019). High levels of AAV vector integration into CRISPR-induced DNA breaks. *Nat. Commun.* 10:4439. 10.1038/s41467-019-12449-2 PMC6769011 31570731

[B97] Harbour J. W. Roberson, Anbunathan H. Onken M. D. Worley L. A. Bowcock A. M. (2013). Recurrent mutations at codon 625 of the splicing factor SF3B1 in uveal melanoma. *Nat. Genet.* 45 133–135. 10.1038/ng.2523 23313955 PMC3789378

[B98] Hardwick S. A. Joglekar A. Flicek P. Frankish A. Tilgner H. U. (2019). Getting the entire message: progress in isoform sequencing. *Front. Genet.* 10:709. 10.3389/fgene.2019.00709 31475029 PMC6706457

[B99] Havens M. A. Duelli D. M. Hastings M. L. (2013). Targeting RNA splicing for disease therapy. *Wiley Interdiscipl. Rev. RNA* 4 247–266. 10.1002/wrna.1158 23512601 PMC3631270

[B100] Havens M. A. Hastings M. L. (2016). Splice-switching antisense oligonucleotides as therapeutic drugs. *Nucleic Acids Res.* 44 6549–6563. 10.1093/nar/gkw533 27288447 PMC5001604

[B101] Hayes G. M. Carrigan P. E. Beck A. M. Miller L. J. (2006). Targeting the RNA splicing machinery as a novel treatment strategy for pancreatic carcinoma. *Cancer Res.* 66 3819–3827. 10.1158/0008-5472.can-05-4065 16585209

[B102] Hazer B. Steinbuchel A. (2007). Increased diversification of polyhydroxyalkanoates by modification reactions for industrial and medical applications. *Appl. Microbiol. Biotechnol.* 74 1–12. 10.1007/s00253-006-0732-8 17146652

[B103] He H. Liyanarachchi S. Akagi K. Nagy R. Li J. Dietrich R. C. (2011). Mutations in U4atac snRNA, a component of the minor spliceosome, in the developmental disorder MOPD I. *Science* 332 238–240. 10.1126/science.1200587 21474760 PMC3380448

[B104] Hermans P. W. van Soolingen D. Bik E. M. de Haas P. E. Dale J. W. van Embden J. D. (1991). Insertion element IS987 from *Mycobacterium bovis* BCG is located in a hot-spot integration region for insertion elements in *Mycobacterium tuberculosis* complex strains. *Infect. Immun.* 59 2695–2705. 10.1128/iai.59.8.2695-2705.1991 1649798 PMC258075

[B105] Hess G. T. Tycko J. Yao D. Bassik M. C. (2017). Methods and applications of CRISPR-mediated base editing in eukaryotic genomes. *Mol. Cell.* 68 26–43. 10.1016/j.molcel.2017.09.029 28985508 PMC5997582

[B106] Ho C. Musa F. Bell C. Walker S. (2015). LDLR gene synonymous mutation c.*1813C*> T results in mRNA splicing variation in a kindred with familial hypercholesterolaemia. *Ann. Clin. Biochem*. 52 680–684. 10.1177/0004563215572702 25624525

[B107] Hong D. Kurzrock R. Kim Y. Woessner R. Younes A. Nemunaitis J. (2015). AZD9150, a next-generation antisense oligonucleotide inhibitor of STAT3 with early evidence of clinical activity in lymphoma and lung cancer. *Sci. Transl. Med.* 7:314ra185. 10.1126/scitranslmed.aac5272 26582900 PMC5279222

[B108] Hosseini E. S. Nikkhah M. Hosseinkhani S. (2019). Cholesterol-rich lipid-mediated nanoparticles boost of transfection efficiency, utilized for gene editing by CRISPR-Cas9. *Int. J. Nanomed.* 14 4353–4366. 10.2147/ijn.s199104 31354265 PMC6579871

[B109] Hsu P. D. Lander E. S. Zhang F. (2014). Development and applications of CRISPR-Cas9 for genome engineering. *Cell* 157 1262–1278. 10.1016/j.cell.2014.05.010 24906146 PMC4343198

[B110] Hunter A. C. (2006). Molecular hurdles in polyfectin design and mechanistic background to polycation induced cytotoxicity. *Adv. Drug Deliv. Rev.* 58 1523–1531. 10.1016/j.addr.2006.09.008 17079050

[B111] Hutton M. Lendon C. L. Rizzu P. Baker M. Froelich S. Houlden H. (1998). Association of missense and 5’-splice-site mutations in tau with the inherited dementia FTDP-17. *Nature* 393 702–705. 9641683 10.1038/31508

[B112] Imielinski M. Berger A. H. Hammerman P. S. Hernandez B. Pugh T. J. Hodis E. (2012). Mapping the hallmarks of lung adenocarcinoma with massively parallel sequencing. *Cell* 150 1107–1120. 22980975 10.1016/j.cell.2012.08.029 PMC3557932

[B113] Ishino Y. Shinagawa H. Makino K. Amemura M. Nakata A. (1987). Nucleotide sequence of the iap gene, responsible for alkaline phosphatase isozyme conversion in *Escherichia coli*, and identification of the gene product. *J. Bacteriol.* 169 5429–5433. 10.1128/jb.169.12.5429-5433.1987 3316184 PMC213968

[B114] Iwatani-Yoshihara M. Ito M. Klein M. G. Yamamoto T. Yonemori K. Tanaka T. (2017). Discovery of allosteric inhibitors targeting the spliceosomal RNA helicase Brr2. *J. Med. Chem.* 60 5759–5771. 10.1021/acs.jmedchem.7b00461 28586220

[B115] Jambhekar S. S. Breen P. (2016). CDs in pharmaceutical formulations I: structure and physicochemical properties, formation of complexes, and types of complex. *Drug Discov. Today* 21 356–362. 10.1016/j.drudis.2015.11.017 26686054

[B116] Jia C. Chen H. Wei M. Chen X. Zhang Y. Cao L. (2017). Gold nanoparticle-based miR155 antagonist macrophage delivery restores the cardiac function in ovariectomized diabetic mouse model. *Int. J. Nanomed.* 12 4963–4979. 10.2147/ijn.s138400 28744126 PMC5513843

[B117] Jinek M. Chylinski K. Fonfara I. Hauer M. Doudna J. A. Charpentier E. (2012). A programmable dual-RNA-guided DNA endonuclease in adaptive bacterial immunity. *Science* 337 816–821. 10.1126/science.1225829 22745249 PMC6286148

[B118] Kaemmerer W. F. (2018). How will the field of gene therapy survive its success? *Bioeng. Transl. Med.* 3 166–177. 10.1002/btm2.10090 30065971 PMC6063870

[B119] Kemaladewi D. U. Bassi P. S. Erwood S. Al-Basha D. Gawlik K. I. Lindsay K. (2019). A mutation-independent approach for muscular dystrophy via upregulation of a modifier gene. *Nature* 572 125–130. 10.1038/s41586-019-1430-x 31341277

[B120] Kemaladewi D. U. Maino E. Hyatt E. Hou H. Ding M. Place K. M. (2017). Correction of a splicing defect in a mouse model of congenital muscular dystrophy type 1A using a homology-directed-repair-independent mechanism. *Nat. Med.* 23 984–989. 10.1038/nm.4367 28714989

[B121] Kennedy P. J. Sousa F. Ferreira D. Pereira C. Nestor M. Oliveira C. (2018). Fab-conjugated PLGA nanoparticles effectively target cancer cells expressing human CD44v6. *Acta Biomater.* 81 208–218. 10.1016/j.actbio.2018.09.043 30267881

[B122] Kim J. Hu C. Moufawad, El Achkar C. Black L. E. Douville J. (2019). Patient-customized oligonucleotide therapy for a rare genetic disease. *N. Engl. J. Med.* 381 1644–1652. 31597037 10.1056/NEJMoa1813279 PMC6961983

[B123] Knott G. J. Doudna J. A. (2018). CRISPR-Cas guides the future of genetic engineering. *Science* 361 866–869. 10.1126/science.aat5011 30166482 PMC6455913

[B124] Kolhar P. Anselmo A. C. Gupta V. Pant K. Prabhakarpandian B. Ruoslahti E. (2013). Using shape effects to target antibody-coated nanoparticles to lung and brain endothelium. *Proc. Natl. Acad. Sci. U.S.A.* 110 10753–10758. 10.1073/pnas.1308345110 23754411 PMC3696781

[B125] Komor A. C. Kim Y. B. Packer M. S. Zuris J. A. Liu D. R. (2016). Programmable editing of a target base in genomic DNA without double-stranded DNA cleavage. *Nature* 533 420–424. 10.1038/nature17946 27096365 PMC4873371

[B126] Krzyszton R. Salem B. Lee D. J. Schwake G. Wagner E. Radler J. O. (2017). Microfluidic self-assembly of folate-targeted monomolecular siRNA-lipid nanoparticles. *Nanoscale* 9 7442–7453. 10.1039/c7nr01593c 28530287

[B127] Lander E. S. (2016). The heroes of CRISPR. *Cell.* 164 18–28. 10.1016/j.cell.2015.12.041 26771483

[B128] Lapuk A. Marr H. Jakkula L. Pedro H. Bhattacharya S. Purdom E. (2010). Exon-level microarray analyses identify alternative splicing programs in breast cancer. *Mol. Cancer Res.* 8 961–974. 10.1158/1541-7786.mcr-09-0528 20605923 PMC2911965

[B129] Lee K. Conboy M. Park H. M. Jiang F. Kim H. J. Dewitt M. A. (2017). Nanoparticle delivery of Cas9 ribonucleoprotein and donor DNA in vivo induces homology-directed DNA repair. *Nat. Biomed. Eng.* 1 889–901. 10.1038/s41551-017-0137-2 29805845 PMC5968829

[B130] Lee S. C. Dvinge H. Kim E. Cho H. Micol J. B. Chung Y. R. (2016). Modulation of splicing catalysis for therapeutic targeting of leukemia with mutations in genes enconding spliceosomal proteins. *Nat. Med.* 22 672–678. 10.1038/nm.4097 27135740 PMC4899191

[B131] Lelli N. Garuti R. Ghisellini M. Tiozzo R. Rolleri M. Aimale V. (1995). Ocurrence of multiple aberrantly spliced mRNAs of the LDL-receptor gene upon a donor splice site mutation that causes familial hypercholesterolemia (FHBenevento). *J. Lipid Res.* 36 1315–1324. 7545204

[B132] Li H. L. Fujimoto N. Sasakawa N. Shirai S. Ohkame T. Sakuma T. (2015). Precise correction of the dystrophin gene in duchenne muscular dystrophy patient induced pluripotent stem cells by TALEN and CRISPR-Cas9. *Stem Cell Rep.* 4 143–154. 10.1016/j.stemcr.2014.10.013 25434822 PMC4297888

[B133] Limeres M. J. Sune-Pou M. Prieto-Sanchez S. Moreno-Castro C. Nusblat A. D. Hernandez-Munain C. (2019). Development and characterization of an improved formulation of cholesteryl oleate-loaded cationic solid-lipid nanoparticles as an efficient non-viral gene delivery system. *Colloids Surf. B Biointerfaces* 184:110533. 10.1016/j.colsurfb.2019.110533 31593829

[B134] Lin J. C. (2017). Therapeutic applications of targeted alternative splicing to cancer treatment. *Int. J. Mol. Sci.* 19:75. 10.3390/ijms19010075 29283381 PMC5796025

[B135] Lopez-Bigas N. Audit B. Ouzounis C. Parra G. Guigo R. (2005). Are splicing mutations the most frequent cause of hereditary disease? *FEBS Lett.* 579 1900–1903. 10.1016/j.febslet.2005.02.047 15792793

[B136] Lorson C. L. Hahnen E. Androphy E. J. Wirth B. (1999). A single nucleotide in the SMN gene regulates splicing and is responsible for spinal muscular atrophy. *Proc. Natl. Acad. Sci. U.SA.* 96 6307–6311. 10.1073/pnas.96.11.6307 10339583 PMC26877

[B137] Lu X. Y. Ciraolo E. Stefenia R. Chen G. Q. Zhang Y. Hirsch E. (2011). Sustained release of PI3K inhibitor from PHA nanoparticles and in vitro growth inhibition of cancer cell lines. *Appl. Microbiol. Biotechnol.* 89 1423–1433. 10.1007/s00253-011-3101-1 21286711

[B138] Maiseyeu A. Mihai G. Roy S. Kherada N. Simonetti O. P. Sen C. K. (2010). Detection of macrophages via paramagnetic vesicles incorporating oxidatively tailored cholesterol ester: an approach for atherosclerosis imaging. *Nanomedicine (Lond)* 5 1341–1356. 10.2217/nnm.10.87 21128718 PMC3098505

[B139] Mali P. Yang L. Esvelt K. M. Aach J. Guell M. DiCarlo J. E. (2013). RNA-guided human genome engineering via Cas9. *Science* 339 823–826. 10.1126/science.1232033 23287722 PMC3712628

[B140] Matera A. G. Wang Z. (2014). A day in the life of the spliceosome. *Nat. Rev. Mol. Cell Biol.* 15 108–121. 10.1038/nrm3742 24452469 PMC4060434

[B141] Mathew V. Wang A. K. (2019). Inotersen: new promise for the treatment of hereditary transthyretin amyloidosis. *Drug Design Dev. Ther.* 13 1515–1525. 10.2147/dddt.s162913 31118583 PMC6507904

[B142] Matos L. Canals I. Dridi L. Choi Y. Prata M. J. Jordan P. (2014). Therapeutic strategies based on modified U1 snRNAs and chaperones for Sanfilippo C splicing mutations. *Orphanet. J. Rare Dis.* 9:180. 10.1186/s13023-014-0180-y PMC4279800 25491247

[B143] Mattioli C. Pianigiani G. De Rocco D. Bianco A. M. Cappelli E. Savoia A. (2014). Unusual splice site mutations disrupt FANCA exon 8 definition. *Biochim. Biophys. Acta* 1842 1052–1058. 10.1016/j.bbadis.2014.03.014 24704046

[B144] McCracken S. Fong N. Yankulov K. Ballantyne S. Pan G. Greenblatt J. (1997). The C-terminal domain of RNA polymerase II couples mRNA processing to transcription. *Nature* 385 357–361. 10.1038/385357a0 9002523

[B145] Meister G. Tuschl T. (2004). Mechanisms of gene silencing by double-stranded RNA. *Nature* 431 343–349. 10.1038/nature02873 15372041

[B146] Mojica F. J. Juez G. Rodriguez-Valera F. (1993). Transcription at different salinities of Haloferax mediterranei sequences adjacent to partially modified PstI sites. *Mol. Microbiol.* 9 613–621. 10.1111/j.1365-2958.1993.tb01721.x 8412707

[B147] Monani U. R. Lorson C. L. Parsons D. W. Prior T. W. Androphy E. J. Burghes A. H. (1999). A single nucleotide difference that alters splicing patterns distinguishes the SMA gene SMN1 from the copy gene SMN2. *Hum. Mol. Genet.* 8 1177–1183. 10.1093/hmg/8.7.1177 10369862

[B148] Montes M. Cloutier A. Sánchez-Hernández N. Michelle L. Lemieux B. Blanchette M. (2012). TCERG1 regulates alternative splicing of Bcl-x gene by modulating the rate of RNAPII transcription. *Mol. Cell. Biol.* 32 751–762. 10.1128/mcb.06255-11 22158966 PMC3272968

[B149] Mukheem A. Muthoosamy K. Manickam S. Sudesh K. Shahabuddin S. Saidur R. (2018). Fabrication and characterization of an electrospun PHA/graphene silver nanocomposite scaffold for antibacterial applications. *Materials (Basel)* 11:1673. 10.3390/ma11091673 30201852 PMC6163631

[B150] Muller R. H. Mader K. Gohla S. (2000). Solid lipid nanoparticles (SLN) for controlled drug delivery – a review of the state of the art. *Eur. J. Pharm. Biopharm.* 50 161–177. 10.1016/s0939-6411(00)00087-4 10840199

[B151] Mustafa S. Devi V. K. Pai R. S. (2017). Effect of PEG and water-soluble chitosan coating on moxifloxacin-loaded PLGA long-circulating nanoparticles. *Drug Deliv. Transl. Res.* 7 27–36. 10.1007/s13346-016-0326-7 27576453

[B152] Naftelberg S. Schor I. E. Ast G. Kornblihtt A. R. (2015). Regulation of alternative splicing through coupling with transcription and chromatin structure. *Annu. Rev. Biochem.* 84 165–198. 10.1146/annurev-biochem-060614-034242 26034889

[B153] Nielsen K. B. Sorensen S. Cartegni L. Corydon T. J. Doktor T. K. Schroeder L. D. (2007). Seemingly neutral polymorphic variants may confer immunity to splicing-inactivating mutations: a synonymous snp in exon 5 of MCAD protects from deleterious mutations in a flanking exonic splicing enhancer. *Am. J. Hum. Genet.* 80 416–432. 10.1086/511992 17273963 PMC1821120

[B154] Nielsen L. R. Schwartz Scheibel E. (1992). Screening for mutations in the gene encoding factor IX. *J. Inher Metab. Dis.* 15 339–341. 10.1007/bf02435971 1357229

[B155] Nik-Zainal S. Davies H. Staaf J. Ramakrishna M. Glodzik D. Zou X. (2016). Landscape of somatic mutations in 560 breast cancer whole-genome sequences. *Nature* 534 47–54. 27135926 10.1038/nature17676 PMC4910866

[B156] Nissim-Rafinia M. Kerem B. (2006). Splicing modulation as a modifier of the CFTR function. *Prog. Mol. Subcell Biol.* 44 233–254. 10.1007/978-3-540-34449-0_10 17076271

[B157] Norcliffe-Kaufmann L. Kaufmann H. (2012). Familial dysautonomia (Riley-Day syndrome): when baroreceptor feedback fails. *Auton Neurosci.* 172 26–30. 10.1016/j.autneu.2012.10.012 23178195

[B158] Osborn M. J. Newby G. A. McElroy A. N. Knipping F. Nielsen S. C. Riddle M. J. (2020). Base editor correction of COL7A1 in recessive dystrophic epidermolysis bullosa patient-derived fibroblasts and iPSCs. *J. Invest. Dermatol.* 140:e335. 10.1016/j.jid.2019.07.701 PMC6983342 31437443

[B159] Ousterout D. G. Kabadi A. M. Thakore P. I. Perez-Pinera P. Brown M. T. Majoros W. H. (2015). Correction of dystrophin expression in cells from Duchenne muscular dystrophy patients through genomic excision of exon 51 by zinc finger nucleases. *Mol. Ther.* 23 523–532. 10.1038/mt.2014.234 25492562 PMC4351462

[B160] Owen N. Zhou H. Malygin A. A. Sangha J. Smith L. D. Muntoni F. (2011). Design principles for bifunctional targeted oligonucleotide enhancers of splicing. *Nucleic Acids Res.* 39 7194–7208. 10.1093/nar/gkr152 21602265 PMC3167598

[B161] Paliwal R. Babu R. J. Palakurthi S. (2014). Nanomedicine scale-up technologies: feasibilities and challenges. *AAPS PharmSciTech.* 15 1527–1534. 10.1208/s12249-014-0177-9 25047256 PMC4245446

[B162] Pan Q. Shai O. Lee L. J. Frey B. J. Blencowe B. J. (2008). Deep surveying of alternative splicing complexity in the human transcriptome by high-throughput sequencing. *Nat. Genet.* 40 1413–1415. 10.1038/ng.259 18978789

[B163] Park E. Pan Z. Zhang Z. Lin L. Xing Y. (2018). The expanding landscape of alternative splicing variation in human populations. *Am. J. Hum. Genet.* 102 11–26. 10.1016/j.ajhg.2017.11.002 29304370 PMC5777382

[B164] Parkesh R. Childs-Disney J. L. Nakamori M. Kumar A. Wang E. Wang T. (2012). Design of a bioactive small molecule that targets the myotonic dystrophy type 1 RNA via an RNA motif-ligand database and chemical similarity searching. *J. Am. Chem. Soc.* 134 4731–4742. 10.1021/ja210088v 22300544 PMC3306011

[B165] Pinotti M. Balestra D. Rizzotto L. Maestri I. Pagani F. Bernardi F. (2009). Rescue of coagulation factor VII function by the U1+5A snRNA. *Blood* 113 6461–6464. 10.1182/blood-2009-03-207613 19387004

[B166] Pinotti M. Rizzotto L. Balestra D. Lewandowska M. A. Cavallari N. Marchetti G. (2008). U1-snRNA-mediated rescue of mRNA processing in severe factor VII deficiency. *Blood* 111 2681–2684. 10.1182/blood-2007-10-117440 18156490

[B167] Planté-Bordeneuve V. Said G. (2011). Familial amyloid polyneuropathy. *Lancet Neurol.* 10 1086–1097. 22094129 10.1016/S1474-4422(11)70246-0

[B168] Porteus M. H. Baltimore D. (2003). Chimeric nucleases stimulate gene targeting in human cells. *Science* 300:763. 10.1126/science.1078395 12730593

[B169] Procter A. M. Phillips J. A. Cooper D. N. (1998). The molecular genetics of growth hormone deficiency. *Hum. Genet.* 103 255–272. 9799079 10.1007/s004390050815

[B170] Qiao C. Liu J. Yang J. Li Y. Weng J. Shao Y. (2016). Enhanced non-inflammasome mediated immune responses by mannosylated zwitterionic-based cationic liposomes for HIV DNA vaccines. *Biomaterials* 85 1–17. 10.1016/j.biomaterials.2016.01.054 26851653

[B171] Radu I. C. Hudita A. Zaharia C. Galateanu B. Iovu H. Tanasa E. V. (2019). Poly(3-hydroxybutyrate-CO-3-hydroxyvalerate) PHBHV biocompatible nanocarriers for 5-FU delivery targeting colorectal cancer. *Drug Deliv.* 26 318–327. 10.1080/10717544.2019.1582729 30896267 PMC6442118

[B172] Ramamoorth M. Narvekar A. (2015). Non viral vectors in gene therapy- an overview. *J. Clin. Diagn Res.* 9 GE01–GE06. 10.7860/JCDR/2015/10443.5394 PMC4347098 25738007

[B173] Rassu G. Soddu E. Posadino A. M. Pintus G. Sarmento B. Giunchedi P. (2017). Nose-to-brain delivery of BACE1 siRNA loaded in solid lipid nanoparticles for Alzheimer’s therapy. *Colloids Surf. B Biointerfaces* 152 296–301. 10.1016/j.colsurfb.2017.01.031 28126681

[B174] Ratni H. Ebeling M. Baird J. Bendels S. Bylund J. Chen K. S. (2018). Discovery of risdiplam, a selective survival of motor neuron-2 (SMN2) gene splicing modifier for the treatment of spinal muscular atrophy (SMA). *J. Med. Chem.* 61 6501–6517. 30044619 10.1021/acs.jmedchem.8b00741

[B175] Rees H. A. Liu D. R. (2018). Base editing: precision chemistry on the genome and transcriptome of living cells. *Nat. Rev. Genet.* 19 770–788. 10.1038/s41576-018-0059-1 30323312 PMC6535181

[B176] Rizk M. Tuzmen S. (2019). Patisiran for the treatment of patients with familial amyloid polyneuropathy. *Drugs Today (Barc)* 55 315–327. 31131842 10.1358/dot.2019.55.5.2958475

[B177] Rodriguez-Martin T. Anthony K. Garcia-Blanco M. A. Mansfield S. G. Anderton B. H. Gallo J. M. (2009). Correction of tau mis-splicing caused by FTDP-17 MAPT mutations by spliceosome-mediated RNA trans-splicing. *Hum. Mol. Genet.* 18 3266–3273. 10.1093/hmg/ddp264 19498037 PMC2722988

[B178] Rodriguez-Martin T. Garcia-Blanco M. A. Mansfield S. G. Grover A. C. Hutton M. Yu Q. (2005). Reprogramming of tau alternative splicing by spliceosome-mediated RNA trans-splicing: implications for tauopathies. *Proc. Natl. Acad. Sci. U.S.A.* 102 15659–15664. 10.1073/pnas.0503150102 16230627 PMC1266082

[B179] Ross S. J. Revenko A. S. Hanson L. L. Ellston R. Staniszewska A. Whalley N. (2017). Targeting KRAS-dependent tumors with AZD4785, a high-affinity therapeutic antisense oligonucleotide inhibitor of KRAS. *Sci. Transl. Med.* 9:eaal5253. 10.1126/scitranslmed.aal5253 28615361

[B180] Ryther R. C. Flynt A. S. Harris B. D. Phillips J. A. III Patton J. G. (2004). GH1 splicing is regulated by multiple enhancers whose mutation produces a dominant-negative GH isoform that can be degraded by allele-specific small interfering RNA (siRNA). *Endocrinology* 145 2988–2996. 10.1210/en.2003-1724 14988388

[B181] Sathasivam K. Neueder A. Gipson T. A. Landles C. Benjamin A. C. Bondulich M. K. (2013). Aberrant splicing of HTT generates the pathogenic exon 1 protein in Huntington disease. *Proc. Natl. Acad. Sci. U.S.A.* 110 2366–2370. 10.1073/pnas.1221891110 23341618 PMC3568346

[B182] Scalet D. Balestra D. Rohban S. Bovolenta M. Perrone D. Bernardi F. (2017). Exploring splicing-switching molecules for seckel syndrome therapy. *Biochim. Biophys. Acta Mol. Basis Dis.* 1863 15–20. 10.1016/j.bbadis.2016.09.011 27639833

[B183] Scalet D. Sacchetto C. Bernardi F. Pinotti M. van de Graaf S. F. J. Balestra D. (2018). The somatic FAH C.*1061C*> A change counteracts the frequent FAH c.1062+5G > A mutation and permits U1snRNA-based splicing correction. *J. Hum. Genet.* 63 683–686. 10.1038/s10038-018-0427-x 29497141 PMC5919117

[B184] Schliecker G. Schmidt C. Fuchs S. Kissel T. (2003). Characterization of a homologous series of D,L-lactic acid oligomers; a mechanistic study on the degradation kinetics in vitro. *Biomaterials* 24 3835–3844. 10.1016/s0142-9612(03)00243-6 12818556

[B185] Schmid F. Hiller T. Korner G. Glaus E. Berger W. Neidhardt J. (2013). A gene therapeutic approach to correct splice defects with modified U1 and U6 snRNPs. *Hum. Gene Ther.* 24 97–104. 10.1089/hum.2012.110 23075156

[B186] Schornack S. Meyer A. Romer P. Jordan T. Lahaye T. (2006). Gene-for-gene-mediated recognition of nuclear-targeted AvrBs3-like bacterial effector proteins. *J. Plant Physiol.* 163 256–272. 10.1016/j.jplph.2005.12.001 16403589

[B187] Scotti M. M. Swanson M. S. (2016). RNA mis-splicing in disease. *Nat. Rev. Genet.* 17 19–32. 10.1038/nrg.2015.3 26593421 PMC5993438

[B188] Sharma V. K. Watts J. K. (2015). Oligonucleotide therapeutics: chemistry, delivery and clinical progress. *Future Med. Chem.* 7 2221–2242. 10.4155/fmc.15.144 26510815

[B189] Shi J. Xu Y. Xu X. Zhu X. Pridgen E. Wu J. (2014). Hybrid lipid-polymer nanoparticles for sustained siRNA delivery and gene silencing. *Nanomedicine* 10 897–900. 24650883 10.1016/j.nano.2014.03.006 PMC4077924

[B190] Siomi M. C. Sato K. Pezic D. Aravin A. A. (2011). PIWI-interacting small RNAs: the vanguard of genome defence. *Nat. Rev. Mol. Cell Biol.* 12 246–258. 10.1038/nrm3089 21427766

[B191] Skordis L. A. Dunckley M. G. Yue B. Eperon I. C. Muntoni F. (2003). Bifunctional antisense oligonucleotides provide a trans-acting splicing enhancer that stimulates SMN2 gene expression in patient fibroblasts. *Proc. Natl. Acad. Sci. U.S.A.* 100 4114–4119. 10.1073/pnas.0633863100 12642665 PMC153057

[B192] Smith J. L. Mou H. Xue W. (2018). Understanding and repurposing CRISPR-mediated alternative splicing. *Genome Biol.* 19:184. 10.1186/s13059-018-1565-3 PMC6219182 30400804

[B193] Smith L. M. Kelleher N. L. Consortium for Top Down P. (2013). Proteoform: a single term describing protein complexity. *Nat. Methods* 10 186–187. 10.1038/nmeth.2369 23443629 PMC4114032

[B194] Somia N. Verma I. M. (2000). Gene therapy: trials and tribulations. *Nat. Rev. Genet.* 1 91–99. 10.1038/35038533 11253666

[B195] Song C. Q. Jiang T. Richter M. Rhym L. H. Koblan L. W. Zafra M. P. (2020). Adenine base editing in an adult mouse model of tyrosinaemia. *Nat. Biomed. Eng.* 4 125–130. 10.1038/s41551-019-0357-8 31740768 PMC6986236

[B196] Song Y. Lou H. H. Boyer J. L. Limberis M. P. Vandenberghe L. H. Hackett N. R. (2009). Functional cystic fibrosis transmembrane conductance regulator expression in cystic fibrosis airway epithelial cells by AAV6.2*-*mediated segmental trans-splicing. *Hum. Gene Ther.* 20 267–281. 10.1089/hum.2008.173 19257851 PMC2855253

[B197] Sreedharan J. Blair I. P. Tripathi V. B. Hu X. Vance C. Rogelj B. (2008). TDP-43 mutations in familial and sporadic amyotrophic lateral sclerosis. *Science* 319 1668–1672. 18309045 10.1126/science.1154584 PMC7116650

[B198] Stadtmauer E. A. Fraietta J. A. Davis M. M. Cohen A. D. Weber K. L. Lancaster E. (2020). CRISPR-engineered T cells in patients with refractory cancer. *Science* 367:eaba7365. 10.1126/science.aba7365 PMC11249135 32029687

[B199] Stenson P. D. Ball E. V. Mort M. Phillips A. D. Shaw K. Cooper D. N. (2012). The human gene mutation database (HGMD) and its exploitation in the fields of personalized genomics and molecular evolution. *Curr. Protoc. Bioinform.* Chapter 1:Unit113. 10.1002/0471250953.bi0113s39 22948725

[B200] Stoilov P. Lin C. H. Damoiseaux R. Nikolic J. Black D. L. (2008). A high-throughput screening strategy identifies cardiotonic steroids as alternative splicing modulators. *Proc. Natl. Acad. Sci. U.S.A.* 105 11218–11223. 10.1073/pnas.0801661105 18678901 PMC2516208

[B201] Strong T. V. Wilkinson D. J. Mansoura M. K. Devor D. C. Henze K. Yang Y. (1993). Expression of an abundant alternatively spliced form of the cystic fibrosis transmembrane conductance regulator (CFTR) gene is not associated with a cAMP-activated chloride conductance. *Hum. Mol. Genet.* 2 225–230. 10.1093/hmg/2.3.225 7684641

[B202] Summerton J. (1999). Morpholino antisense oligomers: the case for an RNase H-independent structural type. *Biochim. Biophys. Acta* 1489 141–158. 10.1016/s0167-4781(99)00150-5 10807004

[B203] Sun L. Wang D. Chen Y. Wang L. Huang P. Li Y. (2017). Core-shell hierarchical mesostructured silica nanoparticles for gene/chemo-synergetic stepwise therapy of multidrug-resistant cancer. *Biomaterials* 133 219–228. 10.1016/j.biomaterials.2017.04.028 28441616

[B204] Sun N. Zhao H. (2013). Transcription activator-like effector nucleases (TALENs): a highly efficient and versatile tool for genome editing. *Biotechnol. Bioeng.* 110 1811–1821. 10.1002/bit.24890 23508559

[B205] Sune-Pou M. Prieto-Sanchez S. Boyero-Corral S. Moreno-Castro C. El Yousfi Y. Sune-Negre J. M. (2017). Targeting splicing in the treatment of human disease. *Genes (Basel)* 8:87. 10.3390/genes8030087 28245575 PMC5368691

[B206] Tabrizi S. J. Leavitt B. R. Landwehrmeyer G. B. Wild E. J. Saft C. Barker R. A. (2019). Targeting huntingtin expression in patients with huntington’s disease. *N. Engl. J. Med.* 380 2307–2316. 31059641 10.1056/NEJMoa1900907

[B207] Tajnik M. Rogalska M. E. Bussani E. Barbon E. Balestra D. Pinotti M. (2016). Molecular basis and therapeutic strategies to rescue factor IX variants that affect splicing and protein function. *PLoS Genet.* 12:e1006082. 10.1371/journal.pgen.1006082 27227676 PMC4882169

[B208] Takata A. Matsumoto N. Kato T. (2017). Genome-wide identification of splicing QTLs in the human brain and their enrichment among schizophrenia-associated loci. *Nat. Commun.* 8:14519. 10.1038/ncomms14519 PMC5333373 28240266

[B209] Taniguchi-Ikeda M. Kobayashi K. Kanagawa M. Yu C. Mori K. Oda T. (2011). Pathogenic exon-trapping by SVA retrotransposon and rescue in Fukuyama muscular dystrophy. *Nature* 478 127–131. 10.1038/nature10456 21979053 PMC3412178

[B210] Tanner G. Glaus E. Barthelmes D. Ader M. Fleischhauer J. Pagani F. (2009). Therapeutic strategy to rescue mutation-induced exon skipping in rhodopsin by adaptation of U1 snRNA. *Hum. Mutat.* 30 255–263. 10.1002/humu.20861 18837008

[B211] Taratula O. Kuzmov A. Shah M. Garbuzenko O. B. Minko T. (2013). Nanostructured lipid carriers as multifunctional nanomedicine platform for pulmonary co-delivery of anticancer drugs and siRNA. *J. Control. Release* 171 349–357. 10.1016/j.jconrel.2013.04.018 23648833 PMC3766401

[B212] Tardy B. L. Tan S. Dam H. H. Ejima H. Blencowe A. Qiao G. G. (2016). Nanoparticles assembled via pH-responsive reversible segregation of CDs in polyrotaxanes. *Nanoscale* 8 15589–15596. 10.1039/c6nr04841b 27509868

[B213] Taylor J. Lee S. C. (2019). Mutations in spliceosome genes and therapeutic opportunities in myeloid malignancies. *Genes Chromosomes Cancer.* 58 889–902. 10.1002/gcc.22784 31334570 PMC6852509

[B214] Thornton C. A. (2014). Myotonic dystrophy. *Neurol. Clin.* 32 705–719. 25037086 10.1016/j.ncl.2014.04.011 PMC4105852

[B215] Tumer Z. (2013). An overview and update of ATP7A mutations leading to menkes disease and occipital horn syndrome. *Hum. Mutat.* 34 417–429. 10.1002/humu.22266 23281160

[B216] Ullrich N. J. Gordon L. B. (2015). Hutchinson-gilford progeria syndrome. *Handb. Clin. Neurol.* 132 249–264. 26564085 10.1016/B978-0-444-62702-5.00018-4

[B217] Uner M. Yener G. (2007). Importance of solid lipid nanoparticles (SLN) in various administration routes and future perspectives. *Int. J. Nanomed.* 2 289–300. PMC2676658 18019829

[B218] van der Woerd W. L. Mulder J. Pagani F. Beuers U. Houwen R. H. van de Graaf S. F. (2015). Analysis of aberrant pre-messenger RNA splicing resulting from mutations in ATP8B1 and efficient in vitro rescue by adapted U1 small nuclear RNA. *Hepatology* 61 1382–1391. 10.1002/hep.27620 25421123

[B219] Verma A. (2018). Recent advances in antisense oligonucleotide therapy in genetic neuromuscular diseases. *Ann. Indian Acad. Neurol.* 21 3–8. 29720791 10.4103/aian.AIAN_298_17 PMC5909143

[B220] Villemaire J. Dion I. Elela S. A. Chabot B. (2003). Reprogramming alternative pre-messenger RNA splicing through the use of protein-binding antisense oligonucleotides. *J. Biol. Chem.* 278 50031–50039. 10.1074/jbc.m308897200 14522969

[B221] Wahl M. C. Will C. L. Luhrmann R. (2009). The spliceosome: design principles of a dynamic RNP machine. *Cell* 136 701–718. 10.1016/j.cell.2009.02.009 19239890

[B222] Wally V. Murauer E. M. Bauer J. W. (2012). Spliceosome-mediated trans-splicing: the therapeutic cut and paste. *J. Invest. Dermatol.* 132 1959–1966. 10.1038/jid.2012.101 22495179

[B223] Wan R. Bai R. Shi Y. (2019). Molecular choreography of pre-mRNA splicing by the spliceosome. *Curr. Opin. Struct. Biol.* 59 124–133. 10.1016/j.sbi.2019.07.010 31476650

[B224] Wang E. T. Sandberg R. Luo S. Khrebtukova I. Zhang L. Mayr C. (2008). Alternative isoform regulation in human tissue transcriptomes. *Nature* 456 470–476. 10.1038/nature07509 18978772 PMC2593745

[B225] Wang X. Yang L. Zhang H. Tian B. Li R. Hou X. (2018). Fluorescent magnetic PEI-PLGA nanoparticles loaded with paclitaxel for concurrent cell imaging, enhanced apoptosis and autophagy in human brain cancer. *Colloids Surf. B Biointerf.* 172 708–717. 10.1016/j.colsurfb.2018.09.033 30245296

[B226] Warf M. B. Nakamori M. Matthys C. M. Thornton C. A. Berglund J. A. (2009). Pentamidine reverses the splicing defects associated with myotonic dystrophy. *Proc. Natl. Acad. Sci. U.S.A.* 106 18551–18556. 10.1073/pnas.0903234106 19822739 PMC2774031

[B227] Wild E. J. Tabrizi S. J. (2017). Therapies targeting DNA and RNA in Huntington’s disease. *Lancet Neurol.* 16 837–847. 10.1016/s1474-4422(17)30280-6 28920889 PMC5604739

[B228] Willyard C. (2018). New human gene tally reignites debate. *Nature* 558 354–355. 10.1038/d41586-018-05462-w 29921859

[B229] Wojtowicz W. M. Wu W. Andre I. Qian B. Baker D. Zipursky S. L. (2007). A vast repertoire of Dscam binding specificities arises from modular interactions of variable Ig domains. *Cell* 130 1134–1145. 10.1016/j.cell.2007.08.026 17889655 PMC2707357

[B230] Wu J. Y. Maniatis T. (1993). Specific interactions between proteins implicated in splice site selection and regulated alternative splicing. *Cell* 75 1061–1070. 10.1016/0092-8674(93)90316-i 8261509

[B231] Xie Q. Jia L. N. Xu H. Y. Hu X. G. Wang W. Jia J. (2016). Fabrication of core-shell PEI/pBMP2-PLGA electrospun scaffold for gene delivery to periodontal ligament stem cells. *Stem Cells Int.* 2016:5385137. 10.1155/2016/5385137 PMC4899599 27313626

[B232] Xu Z. P. Zeng Q. Lu G.-Q. M. Yu A. (2006). Inorganic nanoparticles as carriers for efficient cellular delivery. *Chem. Eng. Sci.* 61 1027–1040. 10.1016/j.ces.2005.06.019

[B233] Yanaizu M. Sakai K. Tosaki Y. Kino Y. Satoh J. (2018). Small nuclear RNA-mediated modulation of splicing reveals a therapeutic strategy for a TREM2 mutation and its post-transcriptional regulation. *Sci. Rep.* 8:6937. 10.1038/s41598-018-25204-2 PMC5931963 29720600

[B234] Yang X. Coulombe-Huntington J. Kang S. Sheynkman G. M. Hao T. Richardson A. (2016). Widespread expansion of protein interaction capabilities by alternative splicing. *Cell* 164 805–817. 26871637 10.1016/j.cell.2016.01.029 PMC4882190

[B235] Yin H. Kanasty R. L. Eltoukhy A. A. Vegas A. J. Dorkin J. R. Anderson D. G. (2014). Non-viral vectors for gene-based therapy. *Nat. Rev. Genet.* 15 541–555. 25022906 10.1038/nrg3763

[B236] Yoshida K. Sanada M. Shiraishi Y. Nowak D. Nagata Y. Yamamoto R. (2011). Frequent pathway mutations of splicing machinery in myelodysplasia. *Nature* 478 64–69. 21909114 10.1038/nature10496

[B237] Yuan J. Ma Y. Huang T. Chen Y. Peng Y. Li B. (2018). Genetic modulation of RNA splicing with a CRISPR-guided cytidine deaminase. *Mol. Cell* 72:e387. 10.1016/j.molcel.2018.09.002 30293782

[B238] Zakharova L. Pashirova T. Kashapov R. Gabdrakhmanov D. Sinyashin O. (2017). *Drug Delivery Mediated by Confined Nanosystems: Structure-Activity Relations and Factors Responsible for the Efficacy of Formulations*, eds E.A.A.M. Grumezescu. Amsterdam: Elsevier.

[B239] Zeitler B. Froelich S. Marlen K. Shivak D. A. Yu Q. Li D. (2019). Allele-selective transcriptional repression of mutant HTT for the treatment of Huntington’s disease. *Nat. Med.* 25 1131–1142. 31263285 10.1038/s41591-019-0478-3

[B240] Zhang J. Shishatskaya E. I. Volova T. G. da Silva L. F. Chen G. Q. (2018). Polyhydroxyalkanoates (PHA) for therapeutic applications. *Mater. Sci. Eng. C Mater. Biol. Appl.* 86 144–150. 10.1016/j.msec.2017.12.035 29525089

[B241] Zhang K. Tang X. Zhang J. Lu W. Lin X. Zhang Y. (2014). PEG-PLGA copolymers: their structure and structure-influenced drug delivery applications. *J. Control. Release* 183 77–86. 10.1016/j.jconrel.2014.03.026 24675377

[B242] Zhou L. Chen Z. Chi W. Yang X. Wang W. Zhang B. (2012). Mono-methoxy-poly(3-hydroxybutyrate-co-4-hydroxybutyrate)-graft-hyper-branched polyethylenimine copolymers for siRNA delivery. *Biomaterials* 33 2334–2344. 10.1016/j.biomaterials.2011.11.060 22154621

[B243] Zhu H. Tucker H. M. Grear K. E. Simpson J. F. Manning A. K. Cupples L. A. (2007). A common polymorphism decreases low-density lipoprotein receptor exon 12 splicing efficiency and associates with increased cholesterol. *Hum. Mol. Genet.* 16 1765–1772. 10.1093/hmg/ddm124 17517690 PMC2361133

[B244] Zuckerman J. E. Gale A. Wu P. Ma R. Davis M. E. (2015). siRNA delivery to the glomerular mesangium using polycationic cyclodextrin nanoparticles containing siRNA. *Nucleic Acid Therap.* 25 53–64. 10.1089/nat.2014.0505 25734248 PMC4376487

